# XomAnnotate: Analysis of Heterogeneous and Complex Exome- A Step towards Translational Medicine

**DOI:** 10.1371/journal.pone.0123569

**Published:** 2015-04-23

**Authors:** Asoke K. Talukder, Shashidhar Ravishankar, Krittika Sasmal, Santhosh Gandham, Jyothsna Prabhukumar, Prahalad H. Achutharao, Debmalya Barh, Francesco Blasi

**Affiliations:** 1 InterpretOmics India Pvt Ltd, #329, 7^th^ Main, HAL 2^nd^ Stage, Indiranagar, Bangalore, 560 008, Karnataka, India; 2 Centre for Genomics and Applied Gene Technology, Institute of Integrative Omics and Applied Biotechnology (IIOAB), Nonakuri, Purba Medinipur, West Bengal, 721172, India; 3 Laboratory of Transcriptional Regulation in Development and Cancer, IFOM (Fondazione Istituto FIRC di Oncologia Molecolare), Milano, Italy; CNR, ITALY

## Abstract

In translational cancer medicine, implicated pathways and the relevant master genes are of focus. Exome's specificity, processing-time, and cost advantage makes it a compelling tool for this purpose. However, analysis of exome lacks reliable combinatory analysis tools and techniques. In this paper we present XomAnnotate – a meta- and functional-analysis software for exome. We compared UnifiedGenotyper, Freebayes, Delly, and Lumpy algorithms that were designed for whole-genome and combined their strengths in XomAnnotate for exome data through meta-analysis to identify comprehensive mutation profile (SNPs/SNVs, short inserts/deletes, and SVs) of patients. The mutation profile is annotated followed by functional analysis through pathway enrichment and network analysis to identify most critical genes and pathways implicated in the disease genesis. The efficacy of the software is verified through MDS and clustering and tested with available 11 familial non-BRCA1/BRCA2 breast cancer exome data. The results showed that the most significantly affected pathways across all samples are cell communication and antigen processing and presentation. ESCO1, HYAL1, RAF1 and PRKCA emerged as the key genes. Network analysis further showed the purine and propanotate metabolism pathways along with RAF1 and PRKCA genes to be master regulators in these patients. Therefore, XomAnnotate is able to use exome data to identify entire mutation landscape, pathways, and the master genes accurately with wide concordance from earlier microarray and whole-genome studies -- making it a suitable biomedical software for using exome in next-generation translational medicine.

**Availability:**

http://www.iomics.in/research/XomAnnotate

## Introduction

Cancer is a disorder caused by variations in the genome [1]. It is a disease not due to individual mutation, or defect in a gene, but of combinations of mutations in genes and their aberrant actions in multiple molecular cascades [2]. The mutations are a combination of point variations and structural variations (SVs) that result into tumorigenesis and its progression [3]. Therefore, the primary objective in cancer genetics is to identify the variants that are responsible for predisposition to cancer [4]. Stratification of cancer will therefore be based on the entire mutation profile of a patient that will include point variations and SVs. The point variations are Single Nucleotide Variants (SNVs) / Single Nucleotide Polymorphisms (SNPs) and short insertions or deletions (indels); SVs in contrast, relate to larger portions of the genome that are deleted, duplicated, inserted, inverted, or translocated within the genome.

One of the most striking features of cancer tissues is their quest for survival that is provided by selective enrichment of variations that give them the edge [5]. Two given cancers may not have any mutations in common; however, they may share the pathways affected by these mutations [2]. The therapeutics of cancer therefore mostly are inhibitors of key genes/proteins of specific pathways that drive the tumorigenesis. For translational and precision medicine, it is therefore necessary to know the entire mutation landscape and implicated pathways for cancer and the master regulatory genes for designing precise personal therapeutics [6, 7].

85% of disease-causing mutations and disease-predisposing SNPs in Mendelian disorders are located in exons and whole exome sequencing provides coverage of more than 95% of the exons in a genome, making it most attractive and effective mechanism to capture clinically important variations [8]. In targeted sequencing only the region under study is scanned making incidental findings less likely. Exome on the other end, indiscriminately includes a holistic approach that helps unearth information critical for personalizing medicine. Furthermore, exonic regions being a small percentage of the whole genome (~2%), takes lesser time to sequence using Next Generation Sequencing (NGS). NGS lowers the turn-around time and is also less expensive; thus making it a more affordable choice for translational medicine [8]. Moreover, in November, 2013, the US Food and Drug Administration (FDA) had approved the NGS platforms for *in vitro* diagnostic (IVD) uses [9].

Excluding synonymous variations, any other variation in the coding or exonic region of a gene is likely to translate into a protein that will function differently [10]. There are few exome analysis tools that perform either SNV or SV analysis. But they do not precisely analyze heterogeneous and complex exome data that includes both SNVs and SVs for translational medicine. Tools such as GATK’s UnifiedGenotyper [11] and Freebayes [12] identify point variations (SNVs and short indels) in the whole genome with their strengths and weaknesses. UnifiedGenotyper uses Bayesian genotype likelihood model to detect SNVs and indels and emits most probable genotypes and allele frequencies in a given dataset [11]. Freebayes in contrast, is able to call more SNVs through a haplotype based variant detection system using a Bayesian model that is capable of modelling multi-allelic loci in a given dataset with non-uniform copy numbers [12]. We used both these tools on exome and whole-genome data and found that they are not biased towards any specific data type (S1 File). On the other hand, tools like GASVPro [13], Delly [14], Lumpy [15] and xHMM [16] are developed for identification of structural variations (SVs). GASVPro, Delly, and Lumpy are designed for whole-genome and they rely on the library size of the paired-end data for identification of structural variations [13–15]. xHMM is the only algorithm designed for exome data that uses Hidden Markov Model (HMM) and Principal Component Analysis (PCA) to discover breakpoints and needs exome datasets for training [16]. Due to wide variability in cancer data, the training of xHMM for cancer exome data is seldom complete leading to incomplete and erroneous results. Similarly, all these SV tools behave differently when used on cancer exome. We analyzed these tools operating on exome data and found that the combination of Delly and Lumpy with some meta-analysis filtering works well for exome data (S2 File).

Similarly, currently available exome specific tools, some of those which use GATK, are also not suitable to be used as standalone tool for exome sample (N = 1) analysis towards development of precision medicine. Among such tools, WEP [17] identifies SNV and deletion/insertion polymorphisms (DIP) but does not address the identification/ prioritization of structural variation. Tools like ExomeCNV [18] and CANOES [19], on the other hand, detect only CNVs and loss of heterozygosity (LOH) based on pile-up or read distribution in the exonic region using a HMM. Unlike ExomeCNV, WEP, and CANOES; TREVA [20] is a combination of several tools that identifies germline susceptibility or somatic variations (SNVs/ indels/ CNVs), where each sample is considered independently. It works on targeted sequencing data. However, TREVA cannot identify translocations.

Keeping all these developments and challenges in mind, we aimed to design an integrative biology analysis software for personalized medicine. We developed ‘XomAnnotate’ (Exome Annotate) using computational algorithms, authored in C/C++ and a functional analysis tool we termed as “XomPathways” in “R”/Bioconductor [21, 22], that generates a complete map of prioritized variants (both point mutations and SVs) derived from exome sequence data generated by GATK UnifiedGenotyper, Freebayes (qualified by SnpEff [23]), Delly, and Lumpy through meta-analysis. We annotated these variations and further used various network analysis and graph theoretical and relational algebra approaches [24, 25] to identify the most critical pathways and master regulatory genes in cancer exome. These pathways are then ranked based on their p-values of being affected. In this paper, we show the efficacy of the XomAnnotate software in analyzing and interpreting highly complex exome dataset for meaningful insight in a series of non BRCA1/2 familial breast cancer.

## Materials and Methods

### Datasets

We used two whole exome sequence datasets from two previous studies available at NCBI SRA (http://www.ncbi.nlm.nih.gov/sra). The first study consists of 11 breast cancer patients (here referred as BCx, x = 1 through 11 samples) from five countries (France, Italy, the Netherlands, Australia, and Spain), comprising of seven families having at least 6 breast cancer cases (between 6 and 10). None of the patients had familial BRCA1/BRCA2 pathogenic mutations and the patients were diagnosed with breast cancer before the age of 60 and no woman was affected with ovarian cancer in these families. The accession numbers of these 11 exome datasets are ERR166303, ERR166304, ERR166307, ERR166308, ERR166310, ERR166312, ERR166315, ERR166330, ERR166333, ERR166335, and ERR166336 [26]. Here we referred these datasets as BC1, BC2, BC3, BC4, BC5, BC6, BC7, BC8, BC9, BC10 and BC11, respectively. The second dataset was selected from 13 healthy individuals with accession numbers ERR031613, ERR031614, ERR031615, ERR031616, ERR031617, ERR031618, ERR031619, ERR031620, ERR031621, ERR031622, ERR031624, ERR031625, and ERR031626 [27]. In this study they are referred as H1, H2, H3, H4, H5, H6, H7, H8, H9, H10, H11, H12 and H13, respectively.

### Analysis

**Fig 1:**
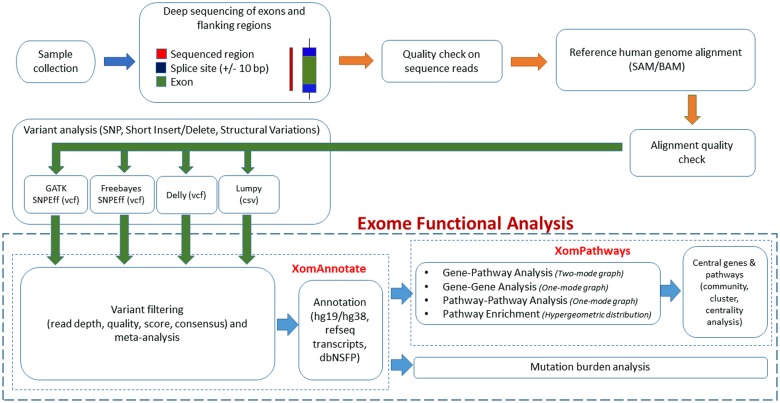
Schematic diagram of XomAnnotate component of iOMICS exome data analysis platform. The diagram shows four main stapes of XomAnnotate software: (i) Variant filtering and meta-analysis, (ii) Annotation of variants, (iii) Pathway enrichment, and (iv) Network analysis.

XomAnnotate component of iOMICS exome data analysis platform (http://interpretomics.co/iomics/) is used for the analysis. The platform is developed at InterpretOmics and the entire analysis software is described in Fig 1.

#### XomAnnotate software of iOMICS

The XomAnnotate software performs four main steps: (i) Variant filtering and Meta-analysis, (ii) Annotation of variants, (iii) Pathway enrichment, and (iv) Network analysis (Fig 1) to identify the most significant variants affecting the most significant pathways and master genes.

#### Variant filtering and meta-analysis

The raw reads (exon sequence +/-10 bp splice sites) of these 24 whole exome samples were first checked to assure high quality of reads using FastQC (http://www.bioinformatics.babraham.ac.uk/projects/fastqc). The best quality reads were then aligned against the human reference genome (hg19) using BowTie Vs.2 [28] and the alignment quality check was carried out using Qualimap (http://qualimap.bioinfo.cipf.es/). The exonic variants were then detected and filtered from the alignment files using UnifiedGenotyper [11] and Freebayes [12] for SNPs/SNVs and indels. Delly [14] and Lumpy [15] were used to detect the SVs.

In XomAnnotate, the meta-analysis is carried out with selected variants that are filtered based on the read depth, quality, score, and consensus of the variants. Since the UnifiedGenotyper and Freebayes are not biased towards any specific data type, for point variations, we therefore took all good quality SNV calls from GATK UnifiedGenotyper and combined them with good quality SNV calls from Freebayes. Variations detected by these tools were passed through SnpEff [23] which qualifies the variation in terms of its impact on the genomic position and gene structure. For structural variations, we combined SVs called by Delly and Lumpy, since as per our analysis, the combination of Delly and Lumpy in addition with some meta-analysis filtering gives good results from complex exome data (see S2 File).

During the meta-analysis, XomAnnotate follows the following rules (configured through a configuration file: see S3 File)

1. An SNV from UnifiedGenotyper having minimum quality score PASSED (50 or above) with a sequencing depth of 20 and above.
2. An SNV from Freebayes will have quality score 50 or above and sequencing depth 20 and above.
3. A unique SNV selected in step 1 or 2 above is checked in dbNSFP database [29]. If this SNV is known (present in the dbNSFP database), the Allele Frequency (AF) for the same SNV is checked. An AF of 0.05 and lower is selected for further downstream processing. An SNV that is deleterious and not available in dbNSFP is considered to be novel and included for downstream analysis.
4. A Lumpy SV will have "Evidence Score" 0.0005 or less.
5. A Delly SV will have minimum of 8 alignments for consensus with a minimum mapping quality 20.
6. A Delly SV will have the FILTER field as PASS.
7. All deleted regions reported as DELETION by Lumpy will be 500 bases or more.
8. A delete must have both breakpoints in the same exon.
9. An inversion must have both breakpoints in exonic regions.
10. A translocation must have both breakpoints in exonic regions. If one of the breakpoints is in exon and the other breakpoint is in intron or intergenic region, the exonic end is included as DELETION.
11. A duplication must have both the breakpoints in the same exon.

#### Annotation

In the annotation step, the selected unique variants derived from the meta-analysis are annotated using a gene table that was obtained through gene collapsing. The gene collapsing is performed to get one entry for a gene that includes all splice variations for that gene even though they might have different RefSeq identifiers. The gene information is taken from hg19 by selecting the "all fields from selected table" in Table Browser of UCSC hg19 genome [30]. This is a TAB separated table that contains all gene fields with their exonic loci. Since in the exome data we are interested only in the coding regions; all non-coding RNA entries with “NR” prefix are removed. The file is now sorted on the basis of loci and selected only entries with “NM” prefix that represent the protein coding entries. All multiple entries of same gene symbol and multiple entries of same exon are merged to a single entry. Once the gene table is obtained, the locus of each variation is taken and the annotation is carried out at the level of exon, amino acid, and nucleotide location with the rsID obtained from dbNSFP [29] and dbSNP (http://www.ncbi.nlm.nih.gov/SNP/).

#### Functional analysis

The list of genes with deleterious variations even after applying above filter were observed to be quite high. Therefore, we focused on the functional analysis of these shortlisted genes in three different ways- (a) pathway enrichment, (c) mutation burden analysis, and (d) centrality analysis using XomPathways component of the XomAnnotate.

#### Pathway enrichment and ranking

In enrichment of cancer pathways, we took deleterious unique genes for point mutations and SVs and 168 cancer and metabolism related KEGG pathways available from Broad Institute [31]. For this step, we used small sample statistical test for categorical variable [32]. The pathway enrichment and its statistical significance is calculated by constructing a two-by-two contingency table and then using Fisher's exact test [33]. For the contingency table, we took the set of mutated genes that are inside and outside of a cancer pathway. The appropriate sampling distribution for such data is hypergeometric [32]. Using this distribution we then measured the probability of "mutated genes" k, from n genes, drawn from a total population N, in a pathway without replacement. If we sample n items without replacement then the p-value is the probability statistic that exactly k genes will be mutated in a pathway. The enriched pathways p-value from this statistical test are then sorted in ascending order which is equivalent to ranking the pathways based on the p-values with most statistically significant pathway first.

#### Mutation burden analysis

In the next part of the enrichment, we looked at the same gene mutation counts differently compared to contingency table and Fisher's test. We wanted to see whether the mutation count or mutation burdens have any relationship with any pathway or cancer. For this analysis, we created a different matrix with our selected 168 KEGG pathways available in MAGENTA tool from Broad Institute [31] with the number of mutated genes in each sample for each of these pathways. The columns in this matrix are divided into two groups viz., healthy and breast cancer patients and elements in the matrix are the deleterious gene counts. In our analysis we took only those genes that contain deleterious point mutations and SVs. We used this matrix and used edgeR [21] of the “R”/Bioconductor statistical package to examine the mutation burdens in different pathways for healthy and cancer groups. We also generated a Multi-Dimensional Scaling (MDS) plot [34] through edgeR [21]. The MDS plot helps us to visually examine the samples and their clusters. The highly significant pathways from this analysis were selected based on their p-value.

#### Network analysis

We used the graph theoretic network analysis as another tool for functional analysis. For network analysis, pathways having at least one mutated gene are selected. From the results of mutations analysis, a binary matrix of dimension Nx(M+1) is created. In this matrix N is the number of KEGG pathways in the rows and M are identified deleterious genes. The first column of this matrix is the total number of genes that are mutated in a particular pathway rest of the columns contain either 1 or 0 to indicate whether a gene is mutated or not in the KEGG pathway. We then generated three adjacency matrices of size NxM, NxN, and MxM from this Nx(M+1) matrix. We used the igraph (http://igraph.org) [35] package in “R” for network analysis. The three undirected acyclic graphs constructed from these three adjacency matrices are: (i) A two-mode bipartite graph of pathway-gene interactions of dimension NxM, (ii) a one-mode graph of pathway-pathway interactions of dimension NxN, where pathways are used as vertex and the participating genes as edges, and (iii) a one-mode graph of gene-gene interactions of dimensions MxM; where genes are used as the vertex, and pathways of their belongingness are used as the edges.

The graph analysis is done by considering the genes and pathways in each sample, which present the mutations, deemed to have significant impact as per the meta-analysis done in the previous step (S4 File). Extensive network analysis is done on these three graphs to discover key genes. We used cluster analysis, community analysis, centrality analysis, and other analysis to examine the network characteristics. From these analyses we identified the central master genes and master pathways implicated.

## Results and Discussion

The 24 exome samples (11 breast cancer and 13 control) [26, 27] were analyzed and the results from the initial variant detection were recorded before performing the meta-analysis. S1 Table shows the statistics of these files.

The all fields table had 44,292 entries in the original UCSC database for hg19. We removed the non-coding regions and collapsed the coding regions. After collapsing, the gene table had 19,945 genes with unique start-end combinations. This gene table was used to annotate the variants.

We ran all 24 exome samples through the XomAnnotate software of iOMICS. The statistics of variants considered for further analysis are given in S2 Table and the annotated results of variants for 11 BC exomes using the gene table are presented in S3 Table (13 healthy samples are not included).

The original paper [26] verified deleterious mutation in 12 genes through wet-lab experiments. These genes are FANCM, WNT8A, CNTROB, CHEK2, SLBP, MAPKAP1, TNFSF8, PTPRF, UBA3, AXIN1, TIMP3, and S1PR3. We manually verified if the XomAnnotate also gives all these 12 genes. Similarly, we compared the output of TREVA pipeline [20] for these 11 BC exomes and compared with the XomAnnotate outcomes. We observed that TREVA gives 11 genes (unfiltered) out of 12, while XomAnnotate gives 12 unfiltered and 8 deleterious genes after filtration. The unfiltered input to XomAnnotate are the raw output from GATK, Freebayes, Delly, Lumpy, and Duppy; whereas, TREVA uses GATK, MuTect etc. as its initial variant detection engine. In fact, the raw variants identified by TREVA can be considered by XomAnnotate for further filtration and downstream analysis. We compared the variant outputs of TREVA and XomAnnotate. We observed that, in case of BC10 [26], the total number of variants detected by TREVA is as high as 19,708. Whereas, XomAnnotate detected only 6,111 and 2,386 variants before and after filtering, respectively. We also observed that 1,978 of the filtered variants of XomAnnotate are common to that of TREVA output. Therefore, efficacy of XomAnnotate is better in variant screening than TREVA.

**Fig 2:**
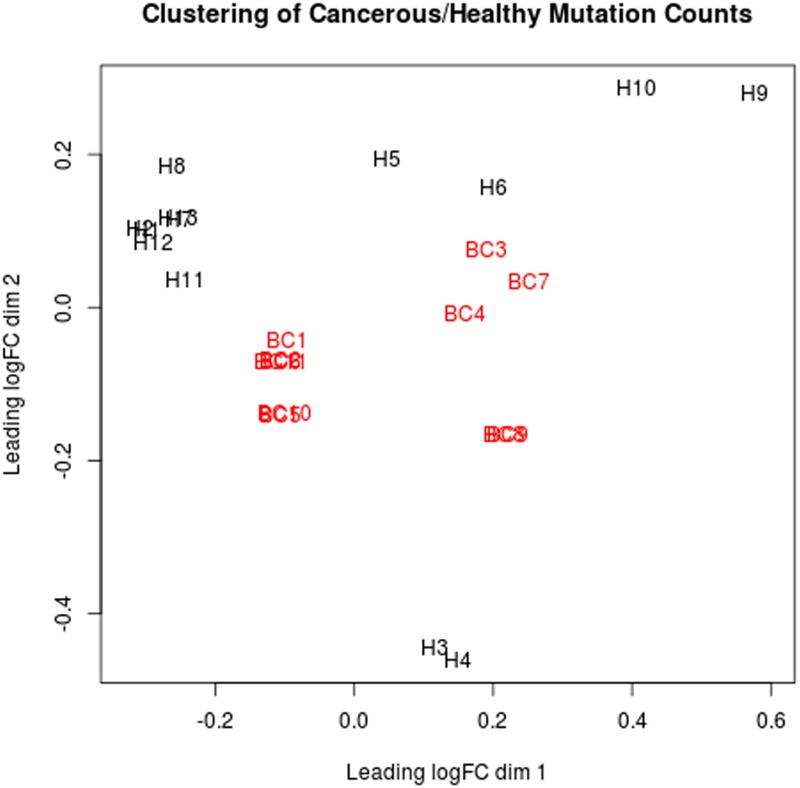
Multidimensional Scaling (MDS) plot of mutation burden of breast cancer and healthy samples. The MDS plot was constructed using entire variation map (mutation count) of the two groups of 11 BC samples against 13 healthy samples. The graph shows distinct clustering of the breast cancer samples (BCx) and the healthy samples (Hx).

To test the efficacy of the XomAnnotate algorithms, we further looked into differential mutation burdens. We calculated the gene counts implicated in each of these selected 168 KEGG pathways [31] for all 24 samples and generated the MDS plot using two groups viz., 11 BC samples and 13 healthy samples’ exomes (Fig 2). The MDS plot used the entire variation map that includes both SNVs/SNPs and SVs. It is evident from the MDS plot (Fig 2) that the mutation counts or mutation burdens of breast cancer (BCx) and the healthy (Hx) samples clustered into two distinct groups indicating that XomAnnotate can precisely detect and predict the pattern of mutations that may influence the development and disease progression. The mutated gene count matrix of both BCx and Hx is given in S4 Table. We found 4 pathways having statistically significant gene mutation burden. These pathways are general ABC transporters, ECM-receptor interaction, cell communication, and metabolism of xenobiotics by cytochrome P450 with p-values being 6.08E-007, 9.18E-007, 6.64E-005, and 0.0002118749, respectively. All these pathways are known to be involved in cancer and dysregulation of ECM pathways are reported to be an early event in breast cancer progression [36–38].

To identify the most significant genes and pathways affected, betweenness-centrality and degree-centrality were identified considering all the genes and pathways that showed mutations. The reasoning behind this step was that, inactivation of any of the central genes or pathways identified by this method would have a significant impact on the progression of the disease [39].

From XomAnnotate pathway enrichment analysis, we found that the most significantly affected pathways amongst all breast cancer samples are cell communication, antigen processing and presentation, and focal adhesion. With a p-value cut off <10e-10; cell communication (for 7 patients), focal adhesion (for 7 patients), and the antigen processing and presentation (for 4 patients) are found to be the most significantly affected pathways. The pathways implicated for each individual patient with their p-values are presented in S5 Table. We compared our results with well-known pathway enrichment tools such as GeneCodis [40] and DAVID [41] and observed that all the pathways identified by XomAnnotate are also enriched by both these tools with similar significance (data not shown).

Deregulation of cell communication and focal adhesion in cancers is not rare [42] and deregulation of antigen processing and presentation and focal adhesion pathways are already reported in breast cancer [38, 43]. MHC class I antigen-processing pathway is regulated by HER-2/neu proto-oncogene status in breast cancer [44]. However, the over-expression of MHC Class II molecules in breast cancer may be due to response to estrogen or cytokines [45].

The pathway-gene interaction was analyzed using bipartite graph theoretic principles. Many large real-world interaction networks in fact are of two-mode networks [46]. In a bipartite graph there are two sets of nodes where every node in one set has a connection to a node on the other set. Example of bipartite graph in the scientific world is the authoring networks, where the authors are linked to the paper they have signed [47]. An example of bipartite graph in the corporate world will be, company board networks, where the board members are linked to the companies they lead [48]. Examples of bipartite graph uses in genomics are comparative genomics [49] or gene-disease relationships [24, 25].

**Fig 3:**
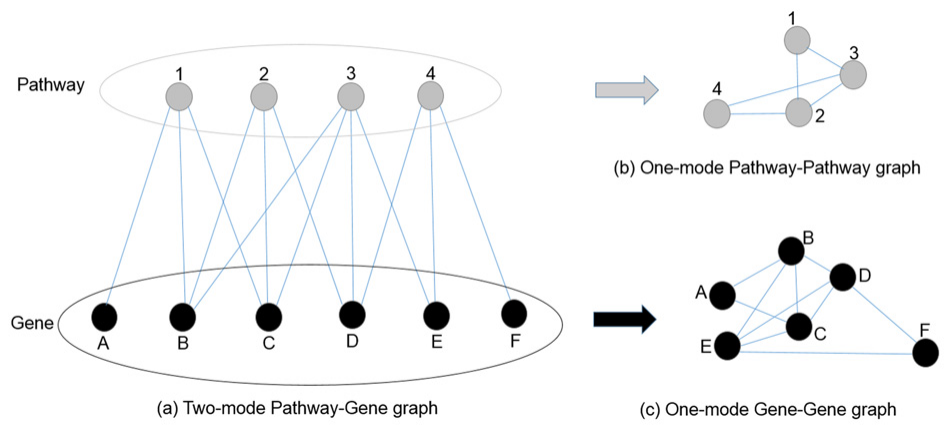
Two-mode and one-mode graph. (a) A bipartite or two-mode graph of pathways and genes. (b) The graph transformed into a one-mode pathway-pathway graph. (c) The graph transformed into a one-mode gene-gene graph.

There is a lack of tools for the analysis of two-mode networks. Also, it is quite complex to conceptualize a two-mode network; therefore, in majority of cases such two-mode networks are transformed into a one-mode network. In a one-mode network all interacting nodes are of same type (S5 File). In such simplified cases, there is an important loss of information; for instance, in Fig 3, pathway-1 and pathway-2 are connected through gene-B and gene-C (Fig 3a). In one-mode network (Fig 3b) we only see that pathway-1 interacts with pathway-2 without any detail of gene-B or gene-C—we even do not get to know the genes’ interactions. We lose the information when there are two different interactions between two pathways [50]. To overcome these challenges we used both one-mode and two-mode gene-pathway graphs.

Graph theory based analysis of pathways and gene interaction networks shows that the most affected KEGG pathways in most of the samples are purine metabolism (BC2, BC6, BC11) and propanoate metabolism (BC5, BC10) (S5 File). Schramm and colleagues reported up-regulation of purine and pyrimidine metabolism in breast cancer that probably increases the rate of cell cycle and therefore the tumor develops the aggressiveness [51]. The other key pathway we obtained, the propanoate metabolism is also shown activated in basal-like residual breast cancers [52]. However, we are not sure about the status of BRCA1/2 in these reported samples. Similarly, most common central genes affected are RAF1 (BC2, BC6, BC11) and PRKCA (BC5, BC10) (S5 File). RAF1 encodes for MAP3K and is known to influence many cellular processes like cell division cycle, apoptosis, cell differentiation, and cell migration. It is also seen that activation of RAF pathways is key for the development and progression of breast cancer [53]. PRKCA is a protein coding gene belonging to PKC (Protein kinase C) family, which is known to be involved in cellular signaling pathways. PKC family members are known to influence many cellular processes such as cell adhesion, cell transformation, cell volume control. Studies have also shown that PRKCA expression is associated with aggressive triple negative breast cancer [54, 55].

Our bipartite graph analysis (S4 File) further confirms that the most significantly affected pathways, as detected by the pathway analysis done earlier, had the most number of interactions and mutations in these pathways. The mutations seen in the affected individuals’ mutation profile show the existence of a pattern, from which it can be proposed that it is necessary for a key set of mutations to occur in a sequence for the occurrence and progression of cancer. This is in line with the existing hypothesis that it requires certain number of mutations for a normal cell to become cancerous [56–58].

From this bipartite graph analysis (S4 File) we found that ESCO1 gene is the mutated gene with the highest interactions. According to Gillis and Pavlidis [59], degree is a measure of multi-functionality, pleiotropy, promiscuity, and hub-ness. This idea can be extended to indicate that ESCO1 plays a significant role in development and progression of cancer due to its high degree of interaction. The three samples (BC2, BC6, and BC11) with the mutated ESCO1 gene, showed a particular mutation N191S (S3 Table). Previous studies show that this rare mutation is known to be associated with familial prostate cancer [60]. Somatic mutations in ESCO1 correlate with endometrial cancer [61] and tamoxifen treatment for breast cancer increases the risk of endometrial cancer [62]. Although there is no known reported association of ESCO1 with breast cancer; since it is involved in sister chromatid pairing [63], ESCO1 may have an association with breast cancer as well.

The analysis also revealed that HYAL1 has mutations and has the highest number of interactions. HYAL1 encodes a lysosomal hyaluronidase. Hyaluronidases degrade hyaluronan, one of the major glycosaminoglycans of the extracellular matrix. Hyaluronan is thought to be involved in cell proliferation, migration and differentiation. It is known that over-expression of HYAL1 correlates with progression and metastasis of breast cancer [64].

The 8 genes (out of the 12 validated genes from the original paper [26]) that we got after XomAnnotate filter, when finally were included in our pathway analysis, we observed that only 4 genes (WNT8A, TNFSF8, PTPRF, S1PR3) are present in our selected 168 KEGG pathways. The graph-based analysis showed that all these genes are associated with 7 statistically significant pathways considering cut off p-value 1.0e-3. Four of them are hedgehog signaling pathway (p-value = 3.67E-007), cytokine-cytokine receptor interaction pathway (p-value = 5.24E-011), cell adhesion molecules (CAMs) pathway (p-value = 2.05E-009), and neuroactive ligand-receptor interaction pathway (p-value = 3.03E-017) (S5 Table and S5 File). Although, all these 4 pathways are associated with cancer [65–68], in our ranking system these first 4 significantly mutated pathways were not identified.

## Conclusions

To the best of our knowledge, there is no tool available to precisely investigate and identify the entire mutation profile of a disease state of an individual patient from exome data. XomAnnotate fills that gap by combining the strengths of the widely used tools like UnifiedGenotyper, Freebayes, Delly, and Lumpy that are used separately for whole-genome for point and structural variations for case-control or cohort studies. We combined all the strengths of these tools through meta-analysis for precise identification and annotation of mutations from exome data for individual patients (N = 1) for translational research. We have also gone beyond the discovery of the variations and attempted to identify the functionality of the mutant genes and its role in the involved pathways.

Our MDS plot (Fig 2) of the total number of variations seen across all samples showed a clear clustering of breast cancer samples and healthy control samples, further proving that XomAnnotate is able to detect mutations accurately which are key to predicting the pattern of mutations which influence the development and progression of cancer. The systems biology part of XomAnnotate that uses multi-mode graphs shows that in spite of heterogeneity in the cancer mutations between patients, the breast cancer data share some common characteristics at its core pathways. Also, this study makes a point that mutations do not occur in a random fashion—the fashion of mutation propagation follows a small-world phenomenon—this is very obvious from the bipartite and one-mode graphs shown in S4 and S5 Files, respectively.

Thus, XomAnnotate is a very effective means of identifying the relationships between the mutations that make up the cumulative basis for disease progression. The ranking of the pathways helps in understanding the relative importance of the disease gene and pathway association, which are hallmarks of the disease and will lead to personalized therapeutics. The confirmation of the impact of the mutations detected by XomAnnotate from previous studies further shows the effectiveness of the methodology. From our analysis on various dataset, spanning the healthy as well as affected spectrum, we conclude that XomAnnotate accurately and with high precision identifies key clusters of genes and pathway, which can have implication with the disease state. We also conclude that XomAnnotate can be used for clinical genomics for exome analysis.

## Supporting Information

S1 File Comparative study between GATK’s Unified Genotyper and Freebayes. There is a large consensus between these two algorithms on SNP calls. Freebayes calls more number of SNP compared to GATK.
(PDF)

S2 File Comparative study of structural variation tools. Comparative study between structural variation detection tools such as Delly, Lumpy, GASVPro and xHMM. xHMM is not very effective for cancer data. Delly is effective for deleted greater than 1k bases; whereas, Lumpy is more sensitive for deletes less than 1k bases.
(PDF)

S3 File Filtering criteria used in XomAnnotate to detect variations of high impact lying within the exonic boundaries. (PDF)

S4 File Pathway-gene bipartite graph analysis. Bipartite graph analysis results of 11 non-BRCA1/BRCA2 breast cancer patients (BC1 to BC11) are represented here. The betweenness and degree centrality pathways and genes are also shown. For each patient, there are four graphs: (a) Bipartite graph plot of vertices and their connections where red dots are the pathways and green dots are genes. (b) Bipartite graph in layered format (two layers) where the upper layer represents pathways and the lower layer representing genes along with their connections and interactions. (c) Histogram of pathway degree distribution. (d) Histogram of gene degree distribution.
(PDF)

S5 File Analysis of Pathway and Gene interaction networks. Results of graph theory based analysis of 11 non-BRCA1/BRCA2 breast cancer patients are presented here. It has included all the genes in KEGG cancer pathways that have been mutated in various samples. The central pathways and genes (betweenness) measured through graph theory are also represented for each breast cancer sample. It also shows the pathway-pathway and the gene-gene graphs that have been constructed for 11 cancer patients. It is observed that the purine metabolism (BC2, BC6, BC11) and propanoate metabolism (BC5, BC10) pathways are most commonly affected and RAF1 (BC2, BC6, BC11) and PRKCA (BC5, BC10) are affected central genes.
(PDF)

S1 Table Downloaded exome data statistics. This table gives information of the raw read count and number of variations detected (unfiltered) by the various tools like GATK’s Unified Genotyper, Freebayes, Delly, and Lumpy.
(PDF)

S2 Table Detailed inventory of samples used for analysis. The table gives detailed information about the samples used, distribution of variations detected by the various tools, the total number of variations considered for meta-analysis and the total number of KEGG pathway genes affected.
(PDF)

S3 Table Annotated results from XomAnnotate. The table gives the results of XomAnnotate analysis of the 11 breast cancer samples considered in this study. The results give information on the position, nucleotide changes, amino acid changes, genes affected, exons affected, and effect of the variations on the structure and function of the gene.
(XLS)

S4 Table The mutated gene count matrix. The table shows the mutation count matrix for all healthy and breast cancer samples considered in this study. The mutation counts indicate the total number of mutations found in all genes present in that pathway for all samples. p-value is calculated based on the count to determine the most significantly affected pathways.
(XLS)

S5 Table Pathway analysis results and list of pathways implicated for each individual patient. The table shows the results of pathway analysis on all 11 breast cancer samples considered in this study. Each page of the spread sheet contains list of pathways of one patient. The pathways are ranked according to their p-values and significance. All the columns from 5 / (F) onwards are showing the point of intersection (genes) between various pathways. It is found that 7 patients have cell communication as the most significant affected pathway. For remaining 4 patients the most significant pathway is antigen processing and presentation. The p-value for all these pathways are < 10e-10.
(XLS)
