# Supplementary material for: XomAnnotate: Analysis of Heterogeneous and Complex Exome- A Step towards Translational Medicine
Source: PLoS One. 2015 Apr 23;10(4):e0123569. doi: 10.1371/journal.pone.0123569 (PMC4408095; doi:10.1371/journal.pone.0123569)
Supplement: S1 File — There is a large consensus between these two algorithms on SNP calls. Freebayes calls more number of SNP compared to GATK. (PDF) [file pone.0123569.s001.pdf]

S1_ File

Comparative study between GATK's Unified Genotyper and Freebayes. There is a large consensus between these two algorithms on SNP calls. Freebays calls more number of SNP compared to GATK.

GATK's UnifiedGenotyper and Freebayes are two of the most widely used variant calling tools for point variations and indels. UnifiedGenotyper is a part of GATK toolkit from Broad Institute. It uses a Bayesian genotype likelihood model to detect SNPs and indels and emit most probable genotypes and allele frequency in a given dataset [1]. Freebayes is a tool that implements a haplotype based variant detection system using a Bayesian model which is capable of modeling multi-allelic loci in a given dataset with non-uniform copy number [2].

Table S1-1: UnifiedGenotyper and Freebayes results

	UnifiedGenotyper	Freebayes
Total variants	24080	129648
Total variants (Q>1000)	8829	20005
Unique variants called(Q> 1000)	1348	12524
Number of High impact variants called	249	284
Time taken	11mins	71mins

The efficacy of the two methods were tested by running them on the same publically available dataset (ERR166310) [3] that is referred to as BC5 in this paper and comparing the variants called and the quality of the variants called. The vcf (variation calling format) outputs of each tool were further annotated using SNPEff [4]. The comparison was done on various factors which include, number of variants called, quality of calls, number of high impact variants detected.

It was found that the total number of variants detected by Freebayes were higher than that of UnifiedGenotyper. The quality distribution (**Fig S1-1, S1-2, S1-3**), that is the ratio of high quality calls to low quality calls, was greater for UnifiedGenotyper. However in terms of absolute numbers, Freebayes detected more number of high quality variants. It was also found that Freebayes was able to detect majority of the variants that UnifiedGenotyper called. The number of unique variants that were detected by Freebayes were much greater than that by UnifiedGenotyper (**Fig S1-S4**).

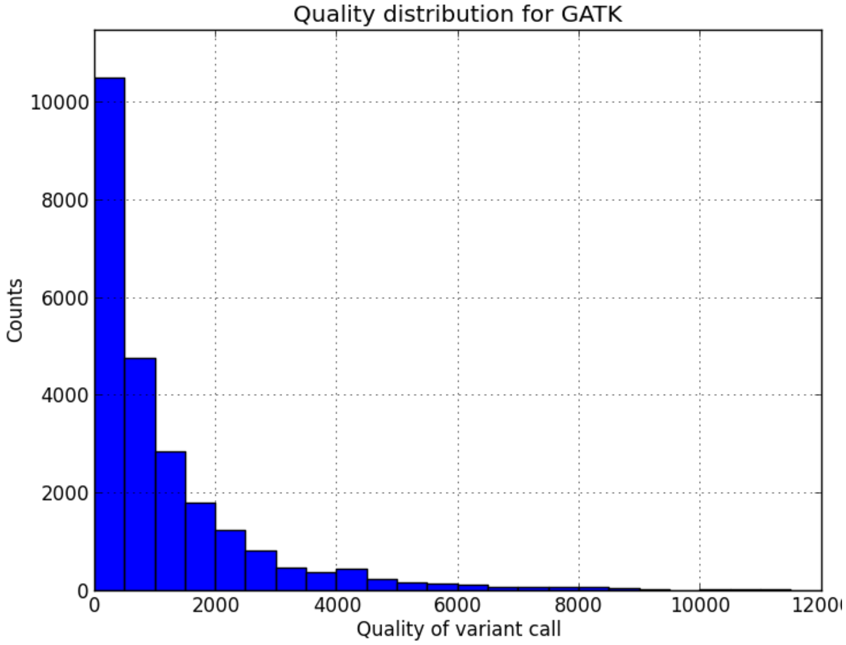

Figure S1-1: Quality score distribution for variants called by GATK

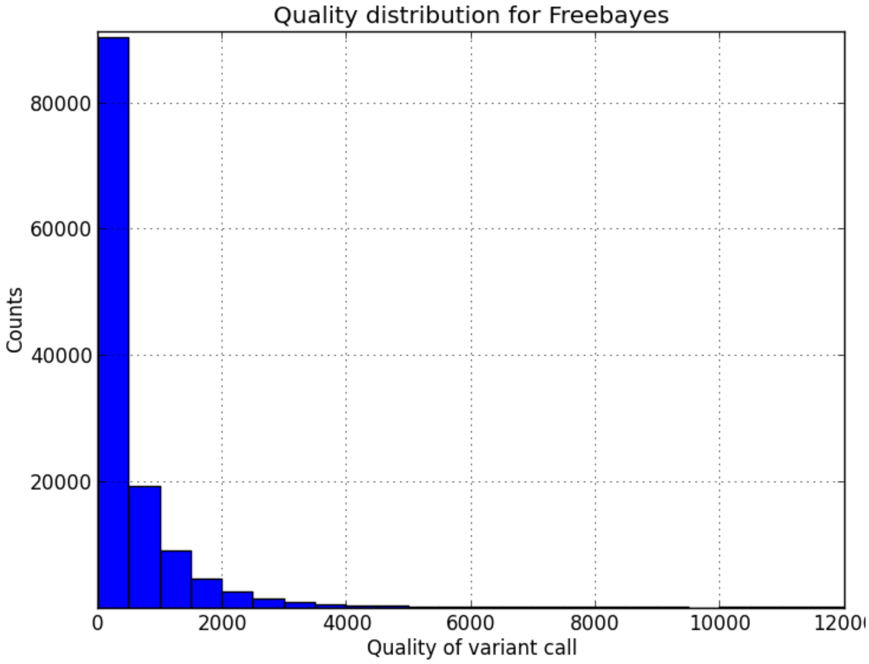

Figure S1-2: Quality score distribution for variants called by Freebayes

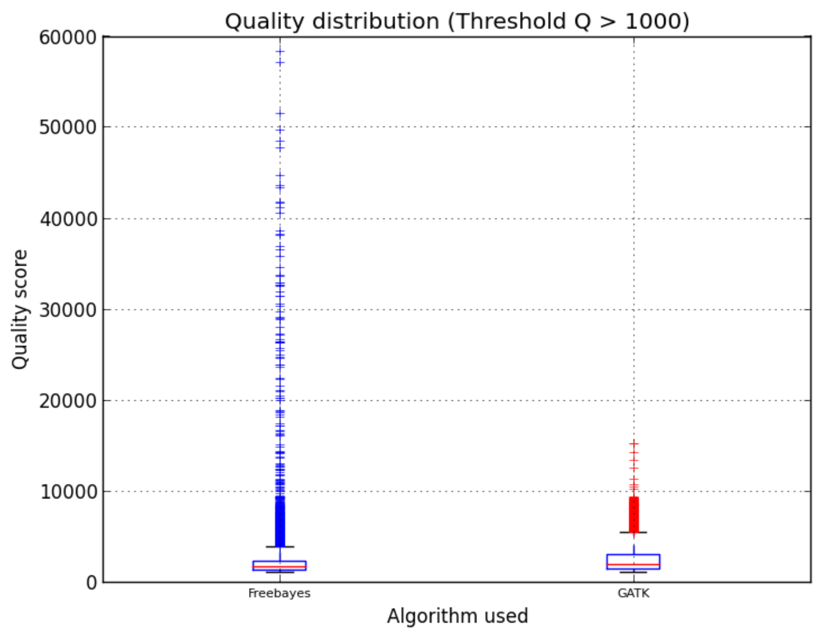

Figure S1-3: Comparison of quality score for variants between GATK and Freebayes

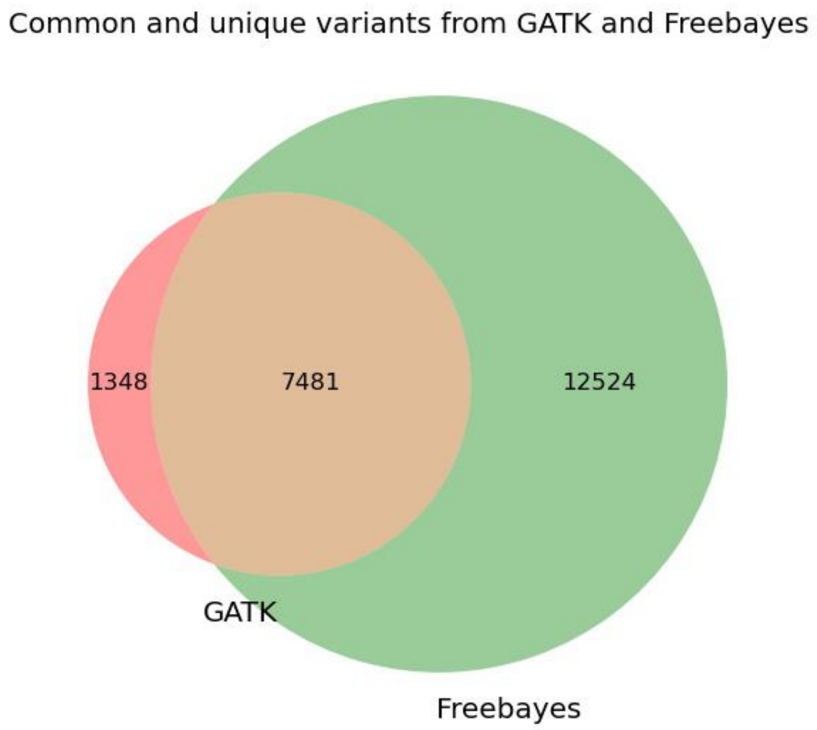

Figure S1-4: Common and unique variants called by GATK and Freebayes

Conclusions

From the tables and statistics it can be seen that even though the quality distribution for GATK is better than Freebayes, in that the ratio to high quality calls to low quality calls is greater. Overall, Freebayes [2] is able to pick out more variants as compared to GATK's UnifiedGenotyper [1]. It may be noted that GATK 2.81 recommends haplotype caller over UnifiedGenotyper, which improves the genotype calls. Freebayes include the haplotype caller as part of its architecture. Therefore, our conclusion is to combine GATK [4] and Freebayes [2] with some filter. We took all SNP discovered by UnifiedGenotyper that are with quality score 50 and above (PASSED). We combined very high quality SNP from Freebayes that are with quality score 2000 and above.

References

1. DePristo M, Banks E, Poplin R, Garimella K, Maguire J, Hartl C, Philippakis A, del Angel G, Rivas MA, Hanna M, McKenna A, Fennell T, Kernytsky A, Sivachenko A, Cibulskis K, Gabriel S, Altshuler D, Daly M (2011). A framework for variation discovery and genotyping using next-generation DNA sequencing data. Nature Genetics.43:491-498.
2. Eric Garrison, Gabor Marth. Haplotype based variant detection from short read sequencing.arXiv:1207.3907.
3. Cingolani P, Platts A, Wang le L, Coon M, Nguyen T, Wang L, Land SJ, Lu X, Ruden DM. Fly (Austin). A program for annotating and predicting the effects of single nucleotide polymorphisms, SnpEff: SNPs in the genome of Drosophila melanogaster strain w1118; iso-2; iso-3.", 2012 Apr-Jun; 6(2):80-92. PMID: 22728672
4. Gracia-Aznarez FJ, Fernandez V, Pita G, Peterlongo P, Dominguez O, de la Hoya M, Duran M, Osorio A, Moreno L, Gonzalez-Neira A, Rosa-Rosa JM,Sinilnikova O, Mazoyer S, Hopper J, Lazaro C, Southey M, Odefrey F, Manoukian S, Catucci I, Caldes T, Lynch HT, Hilbers FS, van Asperen CJ, VasenHF,Goldgar D, Radice P, Devilee P, Benitez J. Wholee xome sequencing suggests much of non BRCA1/BRCA2 familial breast cancer is due to moderate and low penetrance susceptibility alleles. PLos One,8(2),2013.
