# Supplementary material for: XomAnnotate: Analysis of Heterogeneous and Complex Exome- A Step towards Translational Medicine
Source: PLoS One. 2015 Apr 23;10(4):e0123569. doi: 10.1371/journal.pone.0123569 (PMC4408095; doi:10.1371/journal.pone.0123569)
Supplement: S2 File — Comparative study between structural variation detection tools such as Delly, Lumpy, GASVPro and xHMM. xHMM is not very effective for cancer data. Delly is effective for deleted greater than 1k bases; whereas, Lumpy is more sensitive for deletes less than 1k bases. (PDF) [file pone.0123569.s002.pdf]

S2_ File

Comparative study of structural variation tools

Comparative study between structural variation detection tools such as Delly, Lumpy, GASVPro and xHMM. xHMM is not very effective for cancer data. Delly is effective for deleted greater than 1k bases; whereas, Lumpy is more sensitive for deletes less than 1k bases.

We compared the performance of four tools for structural variations of breast cancer exome data. These tools are xHMM [2], GASVPro [6], Delly [4], and Lumpy [5]. xHMM is designed to work on exome data, whereas all other tools were designed to work on whole genome. xHMM is designed for exome data and it uses Hidden Markov Model and Principle Component Analysis to train its core algorithm. GASVPro, Delly, and Lumpy on contrast is specifically designed for whole-genome data.

xHMM was trained on a data-set of 20 exome samples of healthy and cancer (hereditary pheochromocytoma) using publicly available data with id ERR031622, ERR031625, ERR031614, ERR031616, ERR031618, ERR031626, ERR031623, ERR031624, ERR031620, ERR031617, ERR031613, ERR031615, ERR031619, ERR031621, ERR031609, ERR031612, ERR031610, ERR031608, ERR031607, ERR031611 [1]. And was run on 5 HNC unpublished cancer data. 25 results of HNC cancer were randomly selected and manually verified using IGV [3] genome browser. 13 out of the 25 randomly selected SVs were correctly called by xHMM [2]. The variability and heterogeneity is so high in cancer cells that xHMM training is seldom complete. Unlike in other diseases, xHMM accuracy is low for cancer exome data. Therefore, xHMM was not considered for inclusion in XomAnnotate pipeline for cancer translational genomics.

An exome data is created from DNA by synthetically removing the intronic and intergenic regions through NGS chemistry and library preparation. To understand the effectiveness of structural variations tools Delly [4], Lumpy [5], and GASVPro [6] on exome data, all three tools were run on the same dataset, downloaded from NCBI's SRA archives. The sample chosen for the comparative study was from a patient affected with non-BRCA1/BRCA2 breast carcinoma (ERR166310), which is referred as BC5 in the main paper. The sample data was analyzed through iOMICS [www.iomics.in/iomics] Exome-seq pipeline. SV calls from different toolkits were randomly chosen and validated manually using IGV genome browser to identify which of the three methods were better at detecting SVs from whole exome data. Delly uses read pair distribution to identify structural variations and then uses split read analysis to refine the breakpoints [4]. GASVPro uses read pair distribution analysis to identify the

breakpoint, and refines the results by performing a read depth normalization [6]. Lumpy variation detection tool identifies the structural variations by using all three signal, read pair distribution, split read analysis and read depth normalization to arrive at accurate breakpoints for structural variations detected [4].

The comparisons were done separately for the different SVs called, namely deletions, duplications, inversions and translocations. Each call was verified for the breakpoints identified, the pair end distribution and depth of coverage at that given breakpoint, and manually checked to see if there was any concordance with the observations and the results from the different tools. It was seen that all three algorithms were able to identify SVs with high precision. The false discovery rate was however high, because the given dataset was whole exome data and not whole genome data for which the tools are optimized.

1. Deletions

Deletion is part of structural variation where a large portion of the genome is deleted. The length varies from few hundreds of bases to kilo bases. We examined which of the three algorithms (GASV, DELLY, LUMPY) can better identify deletions from whole exome data. All three algorithms were run on the same dataset and ten random calls from GASV, DELLY and LUMPY were visualized in IGV to validate the authenticity and specificity of the deletion called.

Observations

GASV: Table: S2-1

SV start – end	IGV start-end	Genes	Comments
chrX: 55172705-55172910	chrX:55172571-55185645	FAM104B	Although GASV picks up a region smaller than that actually visualized, the call is accurate.
Chr11: 48367420-48367653	Chr11:48367328-48373841	OR4C45	The depth of coverage is quite low, but the 5 deletions can be visualized, although again GASV picks up a smaller regions, it is still accurate.
Chr1: 7889929-7890208	Chr1:7889763-7890309	PER3	In this case the deleted visualized and

			called GASV is of the same length and can be a true call.
Chr8: 6587773-6587950	Chr8: 6587709-6588404	AGPAT5	The call is in an intronic regions, but all reads visualized show the delete, the delete is visualized to extend into exonic region of the gene(exon3)
chrX: 55172665-55172822	chrX:55172571-55185645	FAM104B	GASV identifies a deletion in exon 3 of FAM104B, which can be visualized but the length called by GASV is smaller. This an overlapping region with the first call, yet is identified as a separate call
Chr5: 23527557-23527829	Chr5:23527209-23527959	PRDM9	GASV is able to identify the delete accurately, but again is unable to get the breakpoints correctly(exon11)
Chr17: 45232038-45232382	Chr17:45221258-45232111	CDC27	GASV identifies the wrong breakpoints in this case, although there is a delete in the region, but the breakpoint I identified by GASV is completely wrong.
Chr19: 6387532-6387694	Chr19:6387390-6388353	GTF2F1	GASV identifies a deletion in a low coverage region, although again the region visualized is larger than the variant called.(exon5)
Chr17: 45216190-45216418	Chr17:45216103-45219385	CDC27	GASV identifies a deletion in a low coverage region, although again the region visualized is larger than the variant called
Chr2: 179315078-179315260	Chr2:179314967-179315895	PRKRA	GASV identifies a deletion in a low coverage region, although again the region visualized is larger than the variant called.(exon2)

Delly: Table: S2-2

SV start-end	IGV start-end	Genes	Comments
chr4:110,448,513-110,448,800		SEC24B	There no deletes that are visualized by IGV in the region called by Delly.
chr9:138,054,602-138,054,743		Intergenic	Delly calls a delete in an intragenic region with less than ten read alignments
chr17:1,412,325-1,412,446		INPP5K (intronic)	Delly calls a delete in an intronic region. No deletes can be visualized in this region.
chr8:144,248,682-144,248,811		Intergenic	Delly calls a delete in an intragenic region with less than ten read alignments
chr5:140,208,907-140,238,931	chr5:140,208,907-140,238,931	PCDHA6	Delly identifies a delete in this region which can be confirmed from IGV. The breakpoints are exact.(30000 len)
chr2:88,074,248-88,074,535		RGPD1	One delete in this region can be visualized, as in the case of GASV breakpoints called are smaller
chr11:47,660,374-47,663,942	Chr11:47,660,258-47,664,002	MTCH2	The delete called by Delly can be visualized in IGV and the breakpoints are called correctly (3568 len)
chr11:1,093,090-1,093,289	Chr11:1,092,965-	MUC2	Delly calls a delete in a low

	1,093,437		coverage region, which can be visualized in IGV. The breakpoints are accurate.
chr2:179,306,430-179,307,992	Chr2:179,306,337-179,308,075	PRKRA	Delly identifies a delete in this region which can be confirmed from IGV. The breakpoints are exact (1000 len).
chr17:45,221,343-45,232,038	chr17:45,221,343-45,232,038	CDC27	Delly identifies a delete in this region which can be confirmed from IGV. The breakpoints are exact (10000 len).

Lumpy: Table: S2-3

SV start-end	IGV start-end	Genes	Comments
chr6:26,017,514-26,017,688		HIST1H1A	No deletes can be visualized from this region identified as a delete by Lumpy*
chr15:72,313,076-72,313,304		MY09A	No deletes can be visualized from this region identified as a delete by Lumpy*
chr11:71,614,375-71,614,530		NR_029192	No deletes can be visualized from this region identified as a delete by Lumpy*
chr1:113,231,904-113,232,155		MOV10	No deletes can be visualized from this region identified as a delete by Lumpy*
chr8:124,238,548-124,238,737		C8orf76	No deletes can be visualized from this region identified as a

			delete by Lumpy*
chrX:103,411,796-103,411,997		FAM199X	No deletes can be visualized from this region identified as a delete by Lumpy*
chr2:11,919,637-11,919,795		LPN1	No deletes can be visualized from this region identified as a delete by Lumpy*
chr7:36,662,845-36,662,960		AOAH	No deletes can be visualized from this region identified as a delete by Lumpy*
chr9:86,834,003-86,834,306		Intergenic	No deletes can be visualized from this region identified as a delete by Lumpy*
chr2:9,994,314-9,994,514		TAF1B	No deletes can be visualized from this region identified as a delete by Lumpy*

*In all cases called by Lumpy except the Intergenic one, the insert was reads was greater than the median insert size of 246.

2. Inversions

Inversion is a type of structural variation of the genome where a segment of DNA that is reversed in orientation with respect to the rest of the chromosome. Pericentric inversions include the centromere, whereas paracentric inversions do not. To identify which of the three algorithms (GASV, DELLY, LUMPY) can better identify inversions from whole exome data, all three algorithms were run on the same dataset and random calls from GASV, DELLY and LUMPY were visualized in IGV to validate the authenticity and specificity of the inversion called.

GASV: Table: S2-4

SV start-end	IGV start-end	Genes	Comments
chr5:115,346,859-115,347,309	Chr5:115,346,514-115,351,067	AQPEP (intron)	GASV identifies inversions in the given region, which is seen in IGV.

			The breakpoints indicated by GASV are smaller than the region visualized
chr12:39,859,907-39,860,204	Chr12:39,859,892-39,860,300	Intergenic	GASV identifies an inversion in an Intergenic region, which can be visualized in IGV. The breakpoint found are highly accurate.

Delly: Table: S2-5

SV start-end	IGV start-end	Gene	Comments
chr9:68,421,817-68,429,199	Chr9:68,421,725-68,429,196	Intergenic-LOC642236	Delly identifies inversion in the given region, which can be visualized using IGV and the breakpoints are extremely accurate
chr2:33141320-33141543	Chr233,141,319-33,141,623	LINC00486	Delly identifies an inversion which can be visualized using IGV, the breakpoints are extremely accurate

Lumpy: Table: S2-6

SV start-end	IGV start-end	Genes	Comments
chr5:115,346,580-115,346,891	Chr5:115,346,514-115,351,067	AQPEP	Lumpy identifies inversions in the given region, which is seen in IGV. The breakpoints indicated by Lumpy are smaller than the region visualized
chr12:71,533,260-71,533,458	Chr12:71,533,197-71,533,651	TSPAN8	Lumpy identifies inversions in the given region, which is seen in IGV. The breakpoints indicated by Lumpy are smaller than the region visualized

3. Translocations

Translocation is a type of structural variation where part of a genome breaks and moves to another location within the genome. To identify which of the three algorithms (GASV, DELLY, LUMPY) can

better identify translocation from whole exome data, all three algorithms were run on the same dataset and random calls from GASV, DELLY and LUMPY were visualized in IGV to validate the authenticity and specificity of the translocation called.

Observations

GASV: Table: S2-7

SV start-end	IGV start-end	Genes	Comments
Chr1: 91852620-chr21: 15457354	Chr1:91852900-chr21:15457392	HFM1	GASV identifies a translocation which can be visualized in IGV
chr5:134264138 – chr17: 42075120		PCB02	The region identified by GASV could not be visualized
chr1:109650566 - chr22:30163282	chr1:109650566 - chr22:30163282	UQCR10	GASV identifies a translocation which can be visualized in IGV
chr1:91853070 - chr23 108297795	chr1:91853070 - chr23 108297795	HFM1	GASV identifies a translocation which can be visualized in IGV

Delly: Table: S2-8

SV start-end	IGV start-end	Genes	Comments
chrX: 55172734 – chr18: 65960339			The region identified by Delly could not be visualized
Chr6:6226281 – chr1: 93167673			The region identified by Delly could not be visualized
Chr5:90129535 – chr2: 68368829	Chr5:90129461 – chr2:68368751	GPR98	Delly identifies a translocation which can be visualized in IGV
Chr11:97507818 – chr4:	Chr11:97507849 – chr4:66439408	EPHA5	Delly identifies a

66439504			translocation which can be visualized in IGV

Lumpy: Table: S2-9

SV start-end	IGV start-end	Genes	Comments
chr16:60603640 - chrX:55185586	Chr16: 60603643 – chrX: 55185604	FAM104B	Lumpy identifies a translocation which can be visualized in IGV
chr8:46948143 - chr17:45258974	Chr8:46948135 – chr17:45266511	CDC27	Lumpy identifies a translocation which can be visualized in IGV
chr7:63572539 - chr12:41757470	Chr7:63572765 – chr12:41757479	PDZRN4	Lumpy identifies a translocation which can be visualized in IGV
chr1:91853025 - chrX:108297658	Chr1:91853055 – chrX:108297766	HFM1	Lumpy identifies a translocation which can be visualized in IGV

4. Duplications

Duplication is a type of structural variation, where part of the genome is duplicated and inserted within the genome. Like the other validation tests, we used the same dataset for all three tools.

Observations

GASVPro is unable to detect any duplications

Delly: Table: S2-10

SV start-end	IGV start-end	Genes	Comments
chr2:133,026,690-133,030,657	chr2:133,026,690-133,030,657	Intergenic	Delly calls duplication in intergenic region which can be visualized by IGV.
chr17:33,478,246-33,478,353	chr17:33,478,246-33,478,320	UNC45B	Delly calls duplication in

			intronic region which can be visualized by IGV.

Lumpy: Table: S2-11

SV start-end	IGV start-end	Genes	Comments
chr2:133,026,521-133,026,830	Chr2:133,026,686 -133,030,633	Intergenic	Lumpy finds duplication in the flanking region of the duplicate which can be visualized using IGV
chr17:33,478,074-33,478,322	Chr17:33,478,246-33,478,320	UNC45B	Lumpy calls a duplication in the intronic region which can be visualized by IGV, the breakpoints called are larger than the duplications visualized

Conclusions

For deletions, it was found that, for cases where the deletes were of length <1kb, GASVPro and Lumpy outperformed Delly in identifying true breakpoints as it can be seen from tables (S2-1, S2-2, S2-3). However, when it comes to deletes of length >1kb, Delly is much more accurate with respect to the other two. Of the 30 deletions considered (10 randomly selected from each tool), it was found that GASVPro was able to accurately call 7 deletes of length <1kb, although it did detect 2 deletes of length >1kb, the breakpoints were not correct. Delly was able to identify 4 deletes of length <1kb although these are in the intronic / intergenic regions, 4 deletes >1kb where the breakpoints called extremely accurate. Lumpy identifies 9 deletes <1kb.

For Inversions it was found that, Delly was able to call inversion with greater confidence and accuracy as compared to the other two methods, as seen from table (S2-4, S2-5, and S2-6). Of the 6 inversions considered (2 for each tool) it was found that GASVPro was able to identify both inversion correctly however the breakpoints for one of the calls was not accurate. Delly was able to identify both the inversions with high accuracy with respect to breakpoints. Lumpy, though it was able to identify the region of inversion, the breakpoints were inaccurate.

For translocation, 4 translocation were randomly considered for verification, table(S2-7, S2-8, S2-9) and it was found that Lumpy showed greater accuracy of the three methods, Lumpy was able to correctly call all four translocations where are GASVPro had only 75% success rate and Delly had only a 50% percent success rate.

With respect to duplications however, none of the three methods were able to identify the SV with high confidence. GASVPro does not detect duplications as of yet. Of the two duplications randomly chosen for verification for the other two methods, table (S2-10, S2-11), Delly was able detect both with high precision w.r.t breakpoints, but the regions identified were intronic in nature, Lumpy was able to identify the region, but the breakpoints identified were not accurate.

References

1. Comino-Méndez I, Gracia-Aznárez FJ, Schiavi F, Landa I, Leandro-García LJ, Letón R, Honrado E, Ramos-Medina R, Caronia D, Pita G, Gómez-Graña A, de Cubas AA, Inglada-Pérez L, Maliszewska A, Taschin E, Bobisse S, Pica G, Loli P, Hernández-Lavado R, Díaz JA, Gómez-Morales M, González-Neira A,Roncador G, Rodríguez-Antona C, Benítez J, Mannelli M, Opocher G,Robledo M,Cascón A. Exome sequencing identifies MAX mutations as a cause of hereditary pheochromocytoma. Nature genetics, 43, 663-667,2011.
2. Fromer M, Moran JL,Chambert K,Banks E,Bergen SE,Ruderfer DM,Handsaker RE,McCarroll SA,O'Donovan MC,Owen MJ,Kirov G,Sullivan PF,Hultman CM,Sklar P,Purcell SM.Discovery and Statistical Genotyping of Copy-Number Variation from Whole-Exome Sequencing Depth.The American Journal of Human Genetics,91,597-607,2012.
3. James T Robison, Helga Thorvaldsdottir, Wendy Winckler, Mitchell Guttman, Eric S Lander, Gad Getz and Jill P Mesirov. Integrative genomics viewer. Nature Biotechnology. 29, 24-26. 2011.
4. Rausch T,Zichner T,Schlattl A,Stütz AM,Benes V,Korbel JO,Delly: structural variant discovery by integrated paired-end and split-read analysis, Bioinformatics, Vol 28, i333-i339, 2012.
5. Ryan M. Layer, Ira M. Hall, Aaron R Quinlan. Lumpy: A probabilistic framework for structural variation discovery. ArXiv: 1210.2342v2 .2012.
6. Shihua Zhang, Xuemei Ning, Xiang-Sun Zhang, Identification of functional modules in a PPI network by clique percolation clustering, Computational Biology and Chemistry 30 (2006) 445–451.
