# Supplementary material for: XomAnnotate: Analysis of Heterogeneous and Complex Exome- A Step towards Translational Medicine
Source: PLoS One. 2015 Apr 23;10(4):e0123569. doi: 10.1371/journal.pone.0123569 (PMC4408095; doi:10.1371/journal.pone.0123569)
Supplement: S3 File — (PDF) [file pone.0123569.s003.pdf]

S3 _ File

Filtering criteria used in XomAnnotate to detect variations of high impact lying within the exonic boundaries.

```
# Objective: This is the configuration file for xominer software.
# The xominer software is to mine variations from exome genomic data.
# The xominer program reads the configuration parameters from this file
# to tune its behavior. The name of this file is always <toolname>.cfg.
#
# NOTICE:
# This software is provided "AS IS", without warranty of any kind,
# express or implied, including but not limited to the warranties
# of merchantability,
#
# Fitness for a particular purpose and noninfringement of third party rights.
# in no event shall the authors or copyright holders be liable for any claim,
# damages or other liabilities, whether in an action of contract, or
# otherwise, arising from, out of or in connection with the software
# or the use or other dealings in the software.
#
concordanceCutoff = 8           # The minimum vote to select a locus for SV
qualityThreshold = 20           # Minimum MAPQ threshold for SAM file
EvidenceScore = 0.0005          # Exclude Lumpy record if it is greater
BasesInDelete = 500             # Number of minimum Bases in Lumpy Delete

Effect_Skip = SYNONYMOUS_CODING # Skip all SNP with this Effect
Effect_Impact = MODERATE        # MODERATE & HIGH
UnifiedGenotyper_Quality = 50   # 50 is PASS for GATK UnifiedGenotyper
FreeBayes_Quality = 50          # Only High quality mutations for Freebayes
AlleleFrequency = 0.05          # Only High quality MAF to be included
```
