# Supplementary material for: XomAnnotate: Analysis of Heterogeneous and Complex Exome- A Step towards Translational Medicine
Source: PLoS One. 2015 Apr 23;10(4):e0123569. doi: 10.1371/journal.pone.0123569 (PMC4408095; doi:10.1371/journal.pone.0123569)
Supplement: S4 File — Bipartite graph analysis results of 11 non-BRCA1/BRCA2 breast cancer patients (BC1 to BC11) are represented here. The betweenness and degree centrality pathways and genes are also shown. For each patient, there are four graphs: (a) Bipartite graph plot of vertices and their connections where red dots are the pathways and green dots are genes. (b) Bipartite graph in layered format (two layers) where the upper layer represents pathways and the lower layer representing genes along with their connections and interactions. (c) Histogram of pathway degree distribution. (d) Histogram of gene degree distribution. (PDF) [file pone.0123569.s004.pdf]

S4_File

Pathway-gene bipartite graph analysis

Bipartite graph analysis results of 11 non-BRCA1/BRCA2 breast cancer patients (BC1 to BC11) are represented here. The betweenness and degree centrality pathways and genes are also shown. For each patient, there are four graphs:

(a) Bipartite graph plot of vertices and their connections where red dots are the pathways and green dots are genes. (b) Bipartite graph in layered format (two layers) where the upper layer represents pathways and the lower layer representing genes along with their connections and interactions. (c) Histogram of pathway degree distribution. (d) Histogram of gene degree distribution.

<u>ERR166303 (BC1)</u>

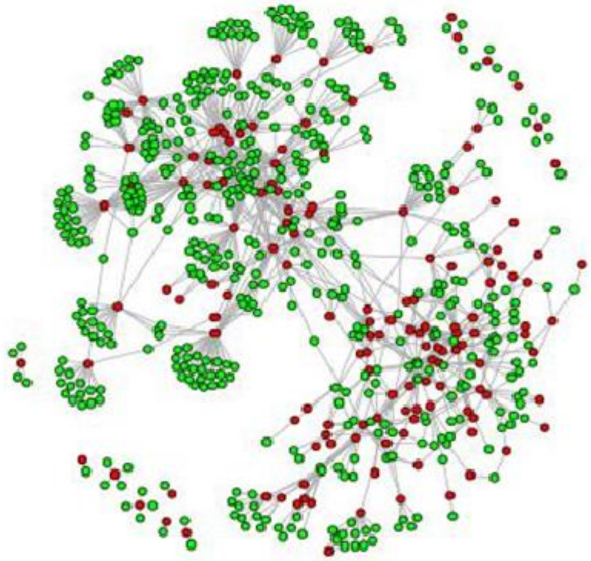

(a) **Bipartite Pathway-Gene Interaction of BC1**

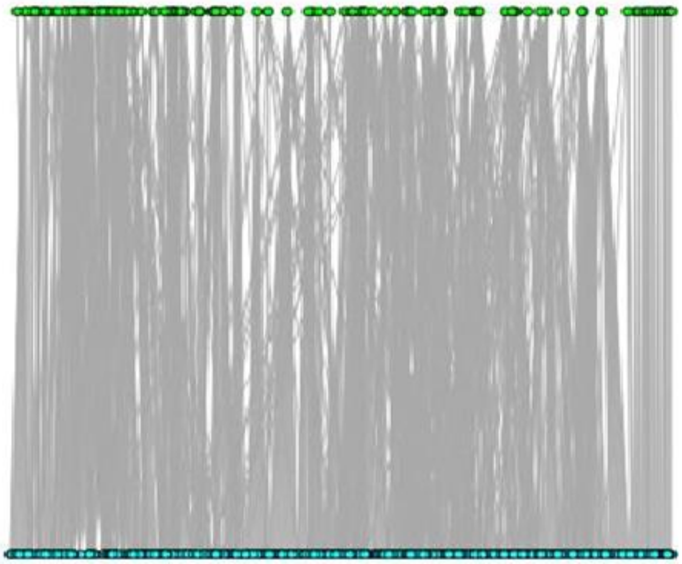

(b) **Pathway-Gene Bipartite Graph**

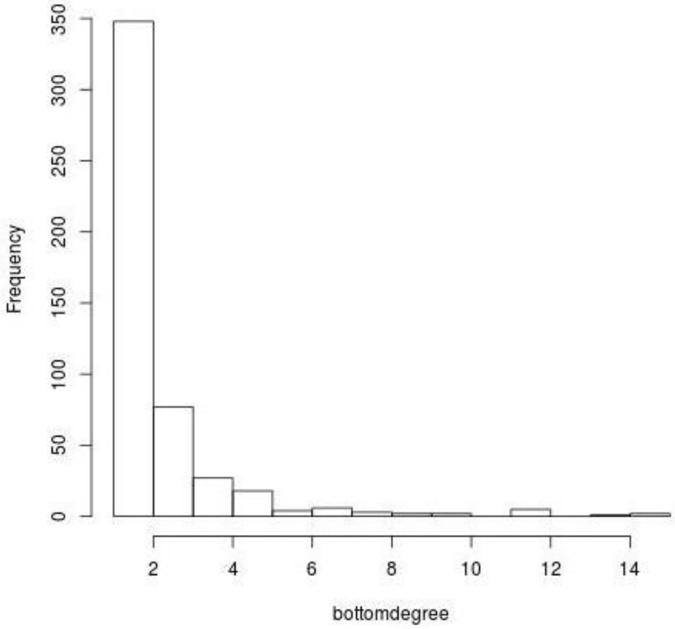

(c) **Bigraph Gene Degree Distribution for BC1**

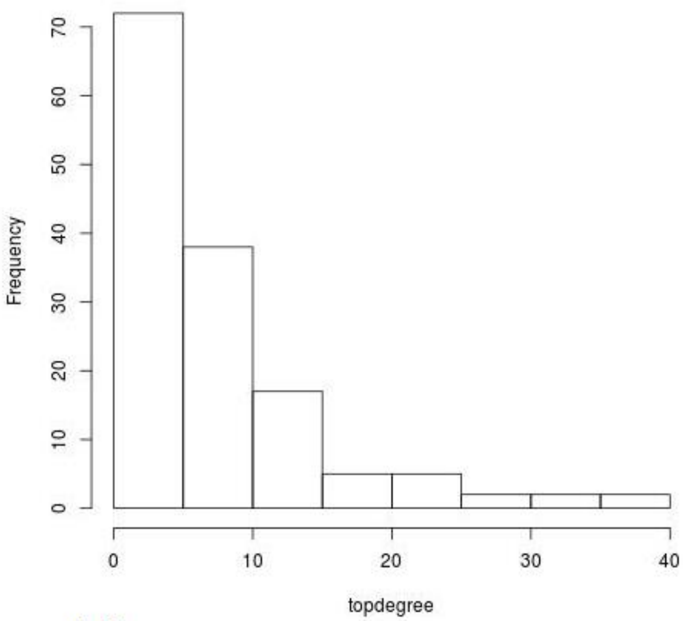

(d) **Bigraph Pathway Degree Distribution for BC1**

ERR166304 (BC2)

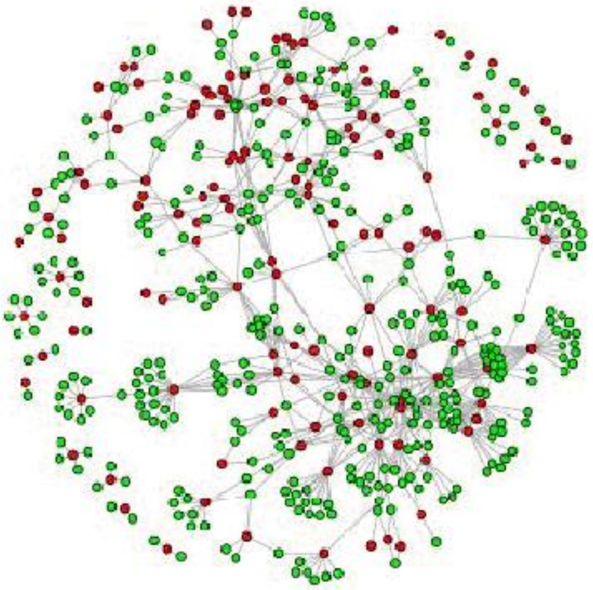

(a) **Bipartite Pathway-Gene Interaction of BC2**

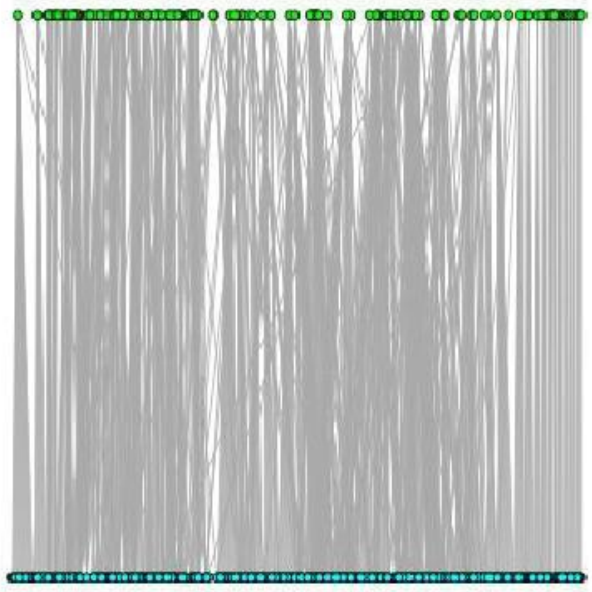

(b) **Pathway-Gene Bipartite Graph**

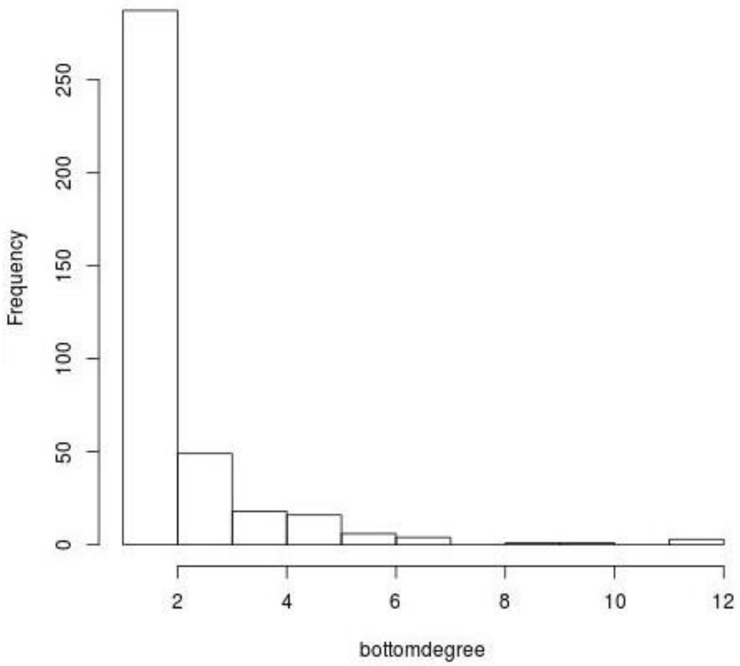

(c) **Bigraph Gene Degree Distribution for BC2**

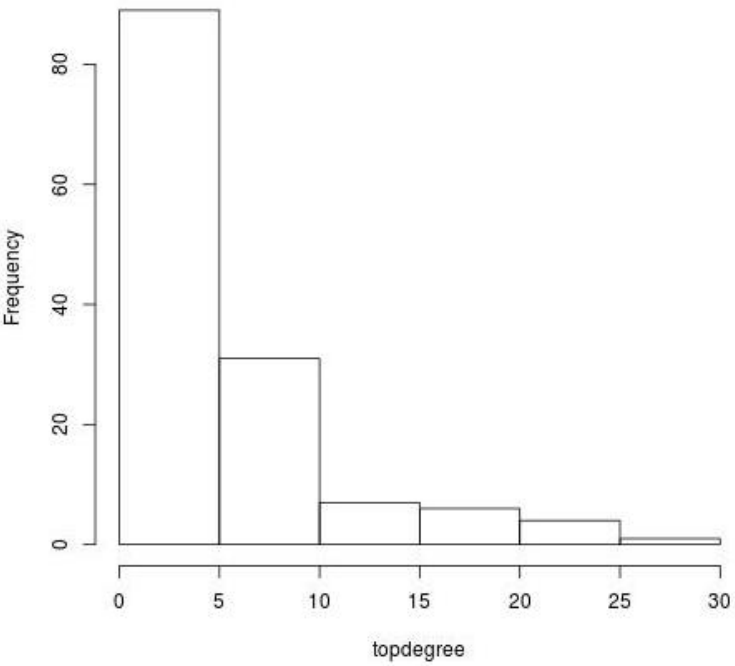

(d) **Bigraph Pathway Degree Distribution for BC2**

ERR166307 (BC3)

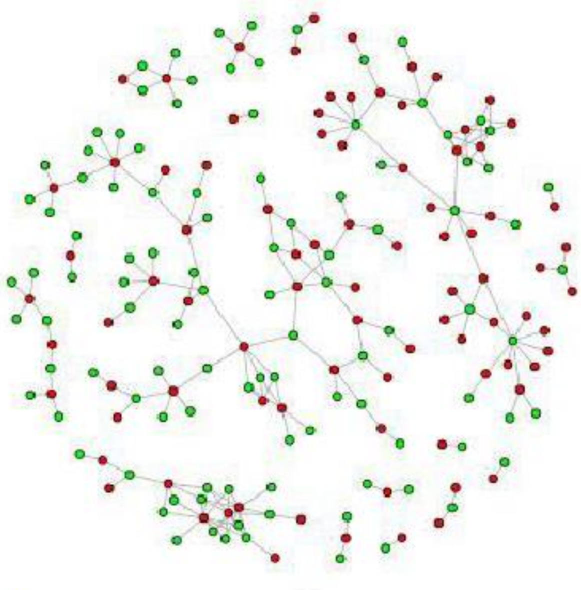

(a) **Bipartite Pathway-Gene Interaction of BC3**

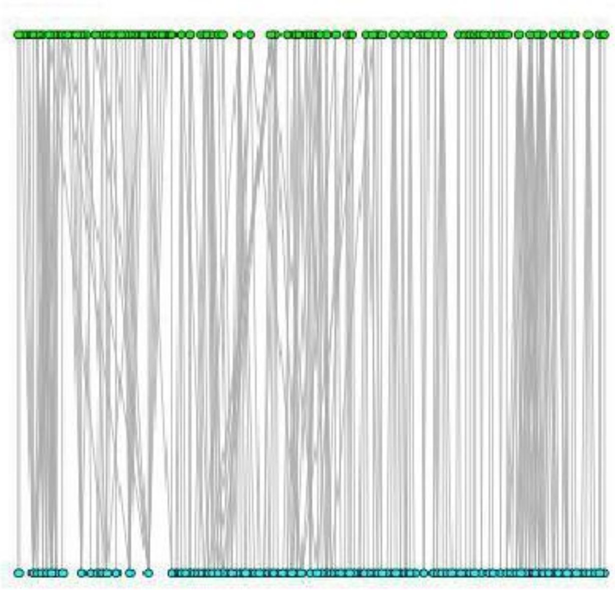

(b) **Pathway-Gene Bipartite Graph**

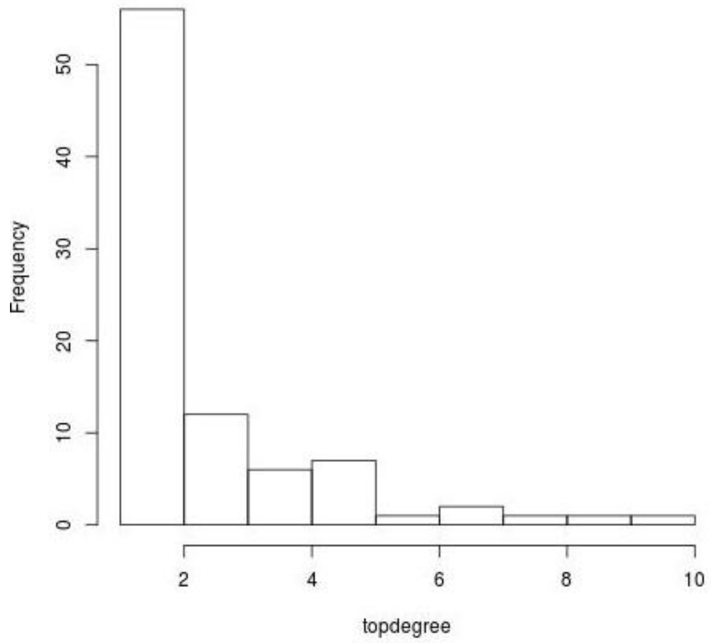

(c) **Bigraph Pathway Degree Distribution for BC3**

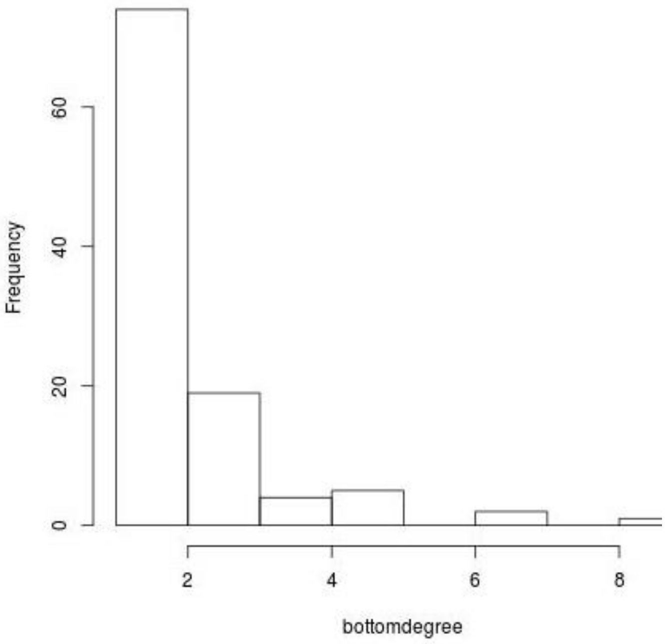

(d) **Bigraph Gene Degree Distribution for BC3**

ERR166308 (BC4)

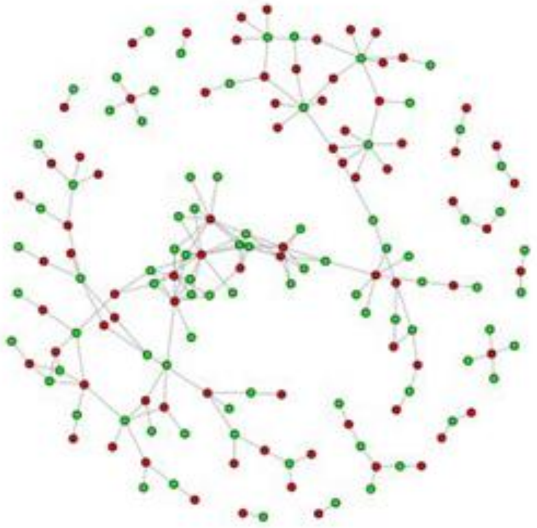

(a) Bipartite Pathway-Gene Interaction of BC4

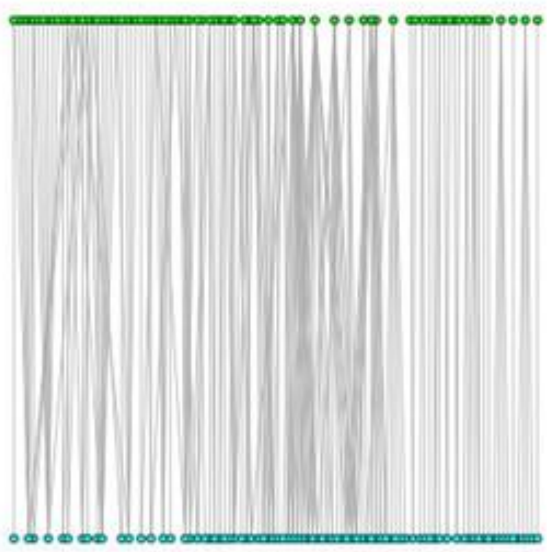

(a) Pathway-Gene Bipartite graph of BC4

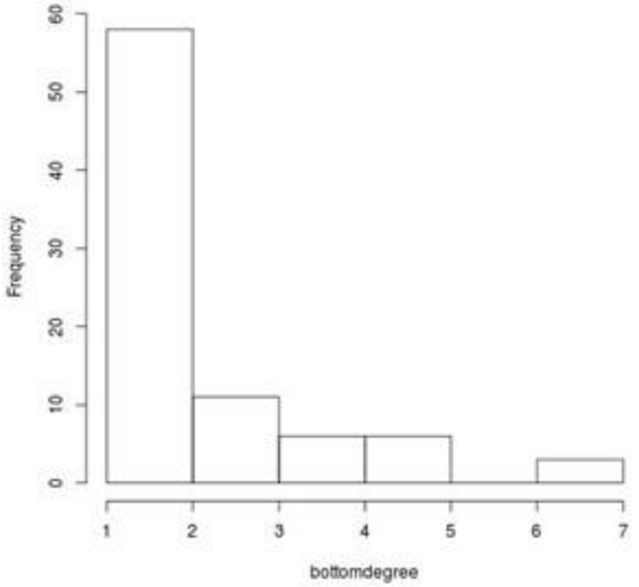

(a) Bigraph Pathway Degree Distribution of BC4

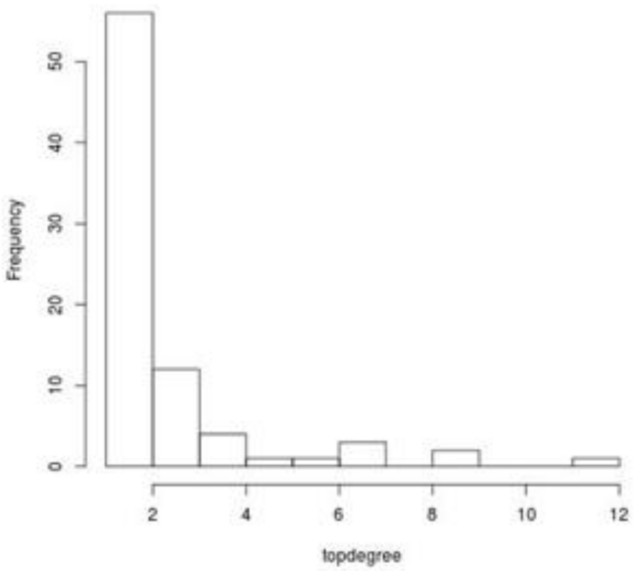

(a) Bigraph Gene Degree Distribution of BC4

ERR166310 (BC5)

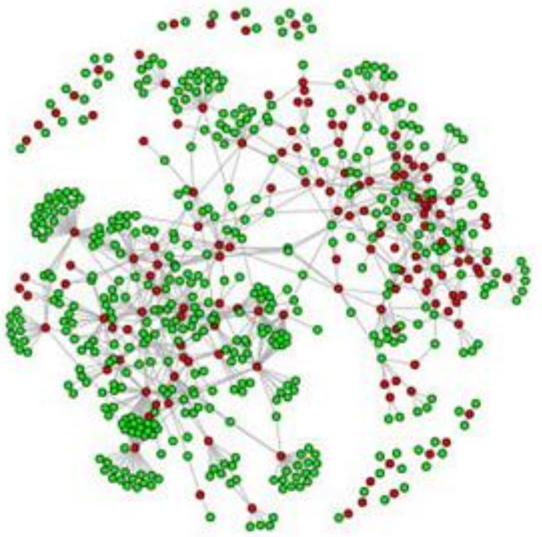

(a) Bipartite Pathway-Gene Interaction of BC5

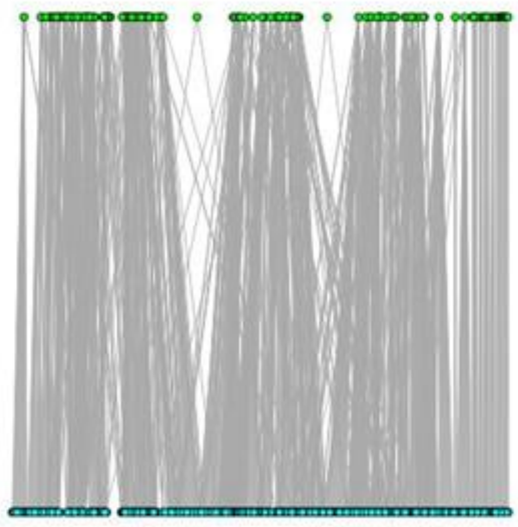

(a) Pathway-Gene Bipartite graph of BC5

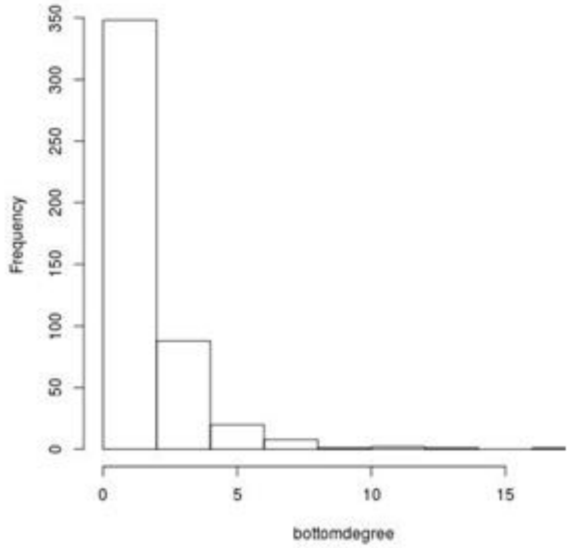

(a) Bigraph Pathway Degree Distribution of BC5

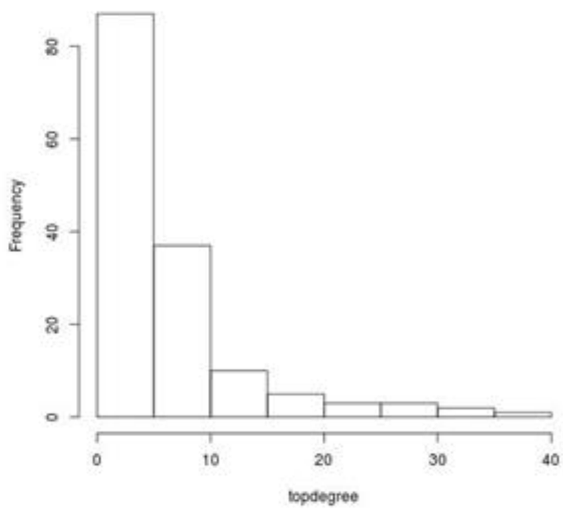

(a) Bigraph Gene Degree Distribution of BC5

ERR166312 (BC6)

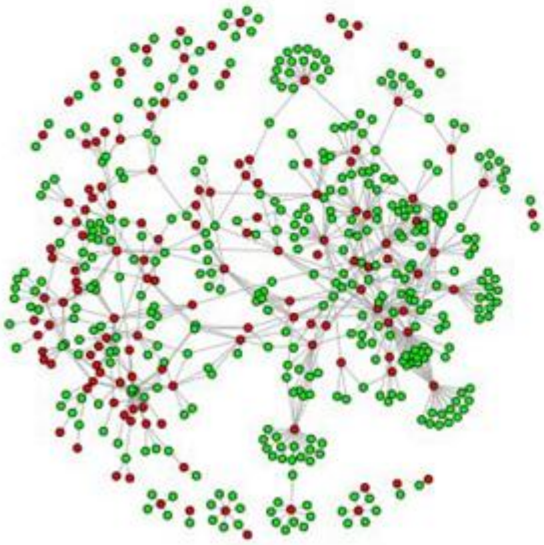

(a) Bipartite Pathway-Gene Interaction of BC6

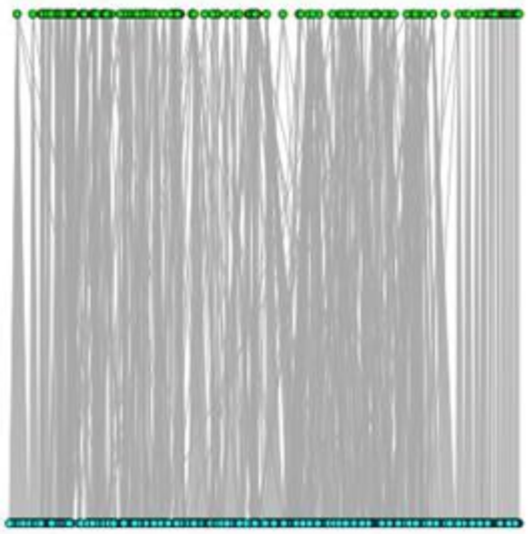

(a) Pathway-Gene Bipartite graph of BC6

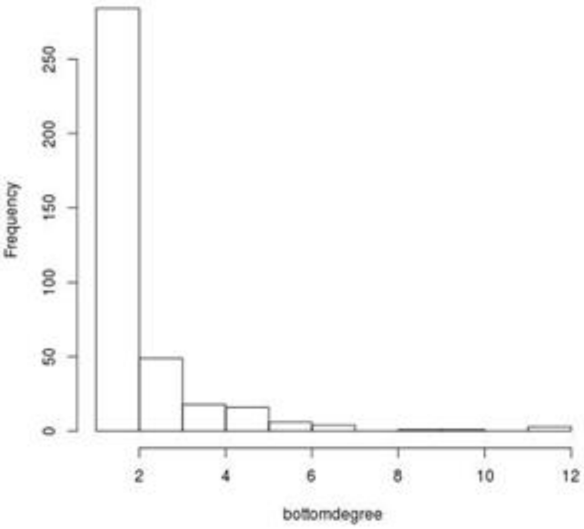

(a) Bigraph Pathway Degree Distribution of BC6

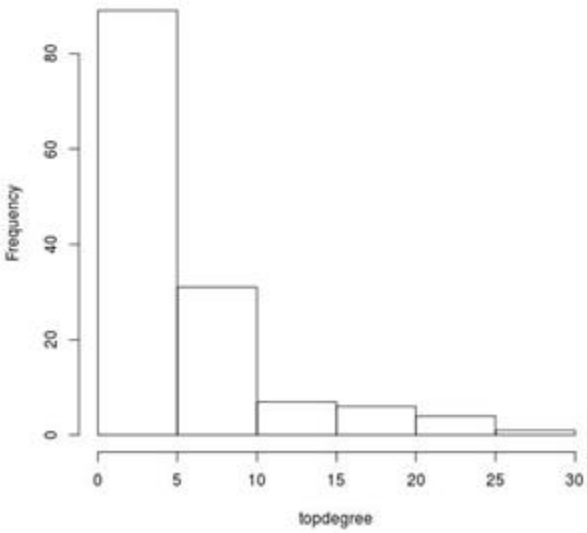

(a) Bigraph Gene Degree Distribution of BC6

ERR166315 (BC7)

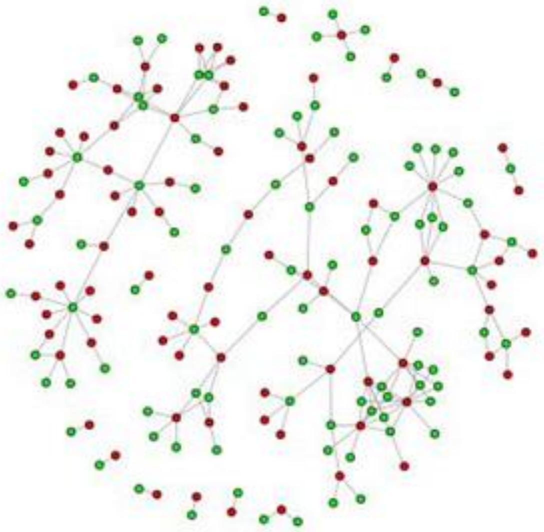

(a) Bipartite Pathway-Gene Interaction of BC7

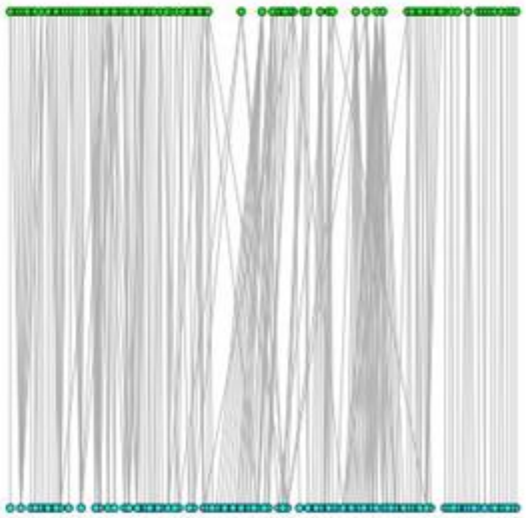

(a) Pathway-Gene Bipartite graph of BC7

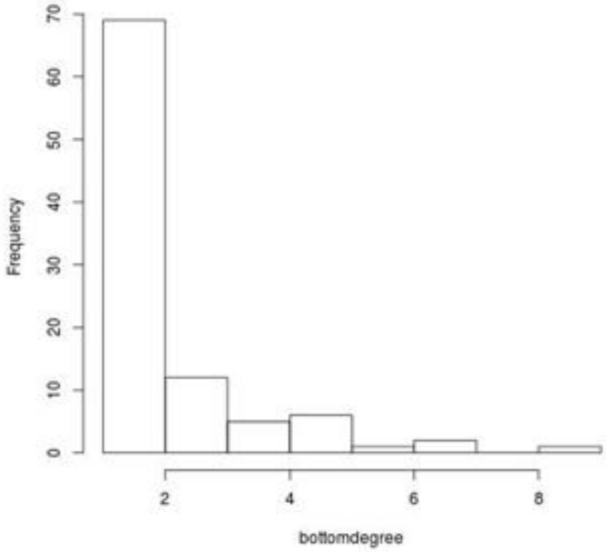

(a) Bigraph Pathway Degree Distribution of BC7

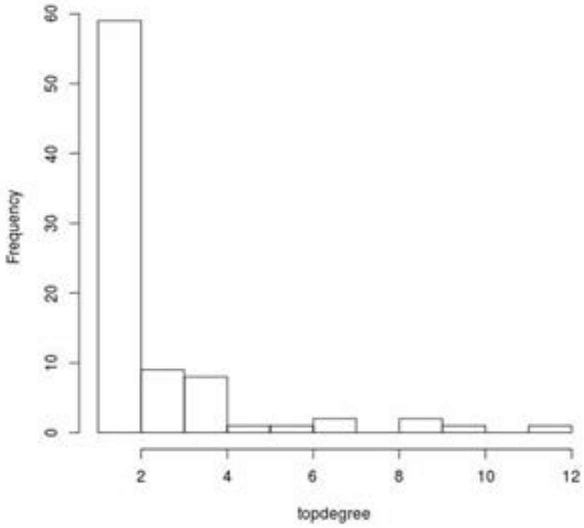

(a) Bigraph Gene Degree Distribution of BC7

ERR166330 (BC8)

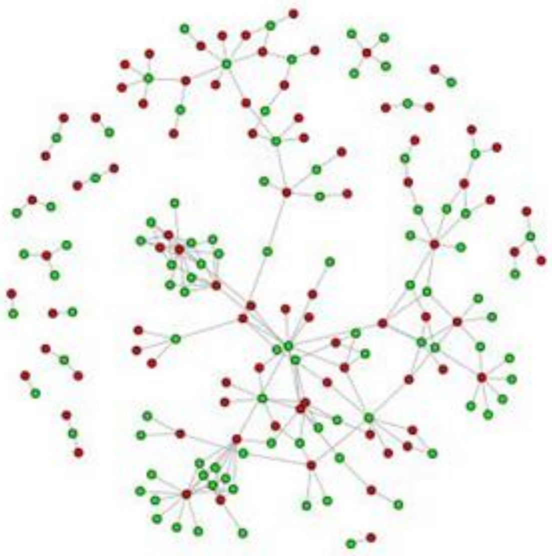

(a) Bipartite Pathway-Gene Interaction of BC8

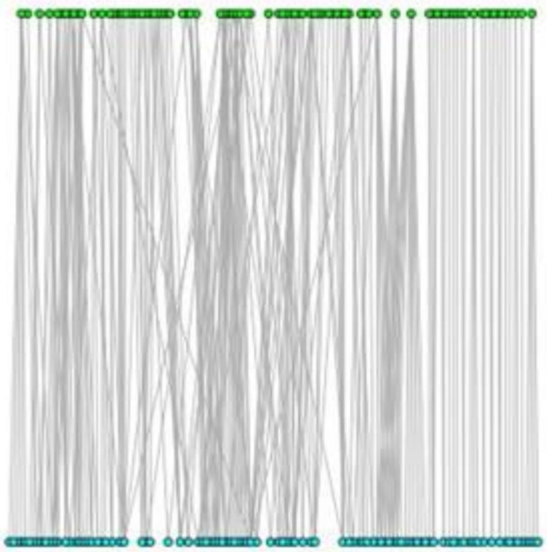

(a) Pathway-Gene Bipartite graph of BC8

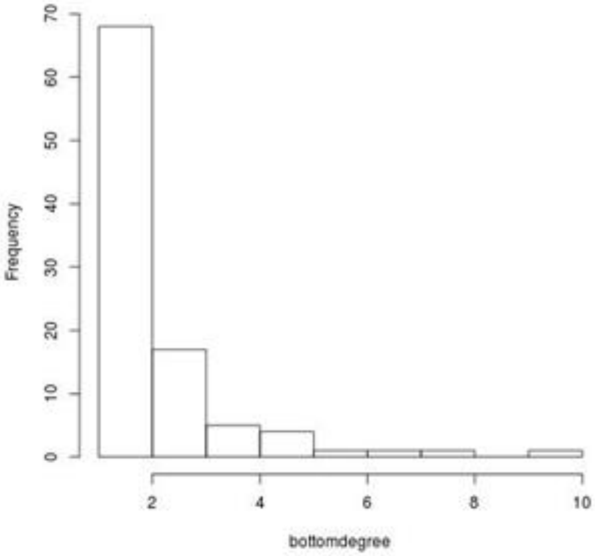

(a) Bigraph Pathway Degree Distribution of BC8

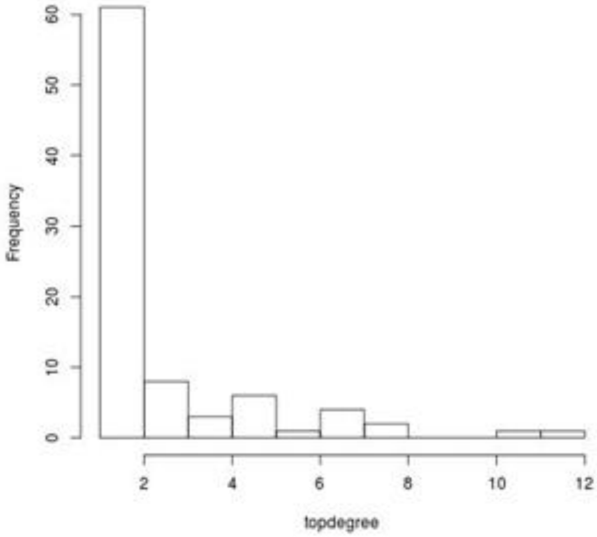

(a) Bigraph Gene Degree Distribution of BC8

ERR166333 (BC9)

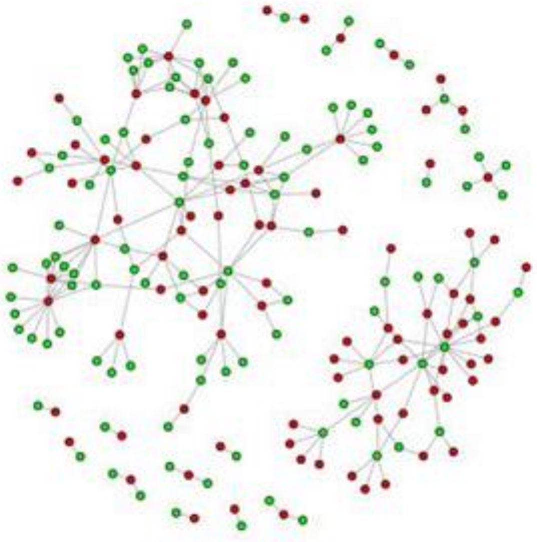

(a) Bipartite Pathway-Gene Interaction of BC9

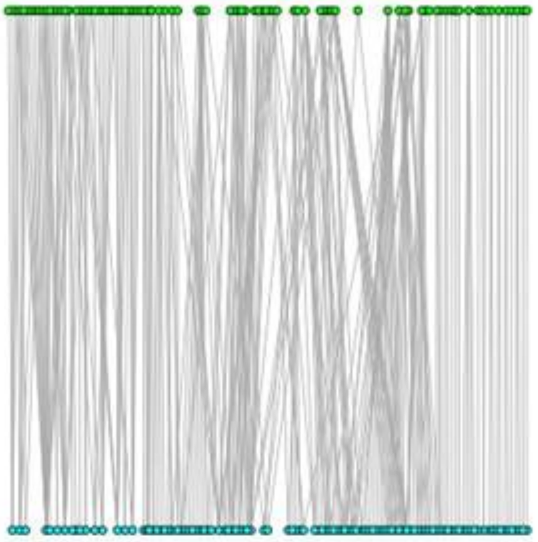

(a) Pathway-Gene Bipartite graph of BC9

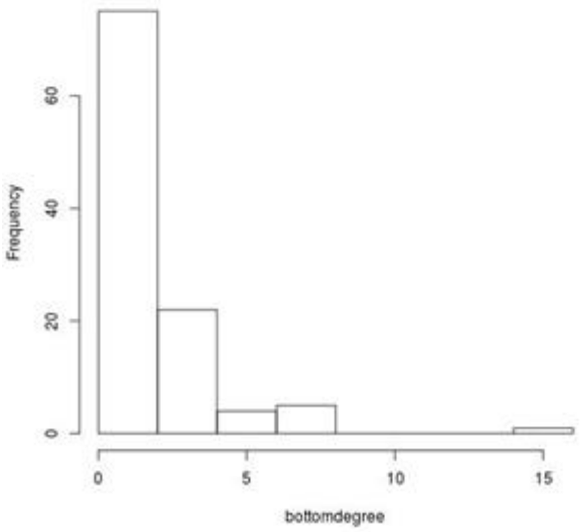

(a) Bigraph Pathway Degree Distribution of BC9

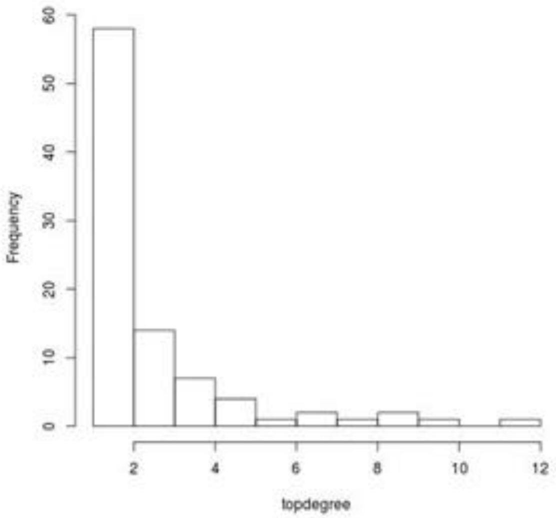

(a) Bigraph Gene Degree Distribution of BC9

ERR166335 (BC10)

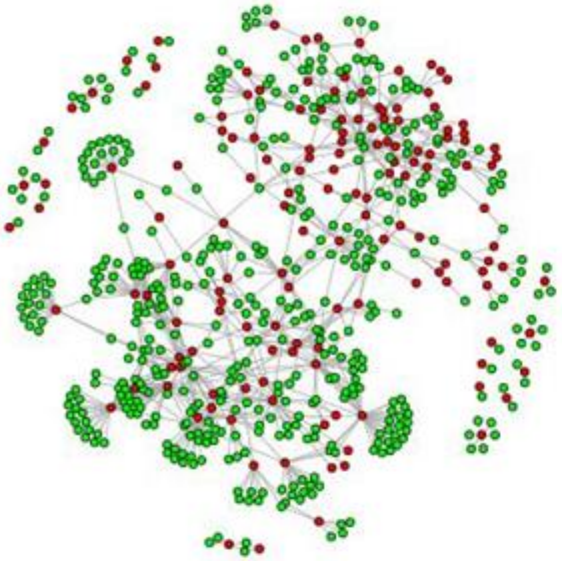

(a) Bipartite Pathway-Gene Interaction of BC10

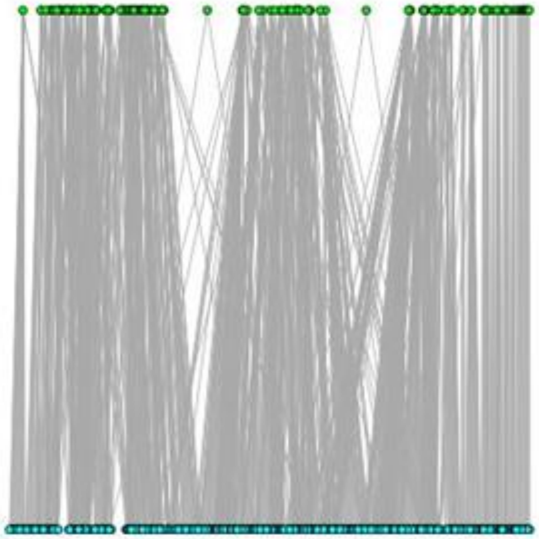

(a) Pathway-Gene Bipartite graph of BC10

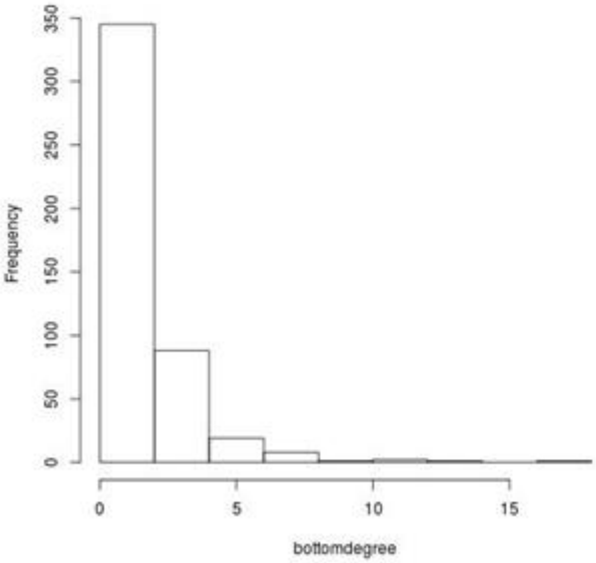

(a) Bigraph Pathway Degree Distribution of BC10

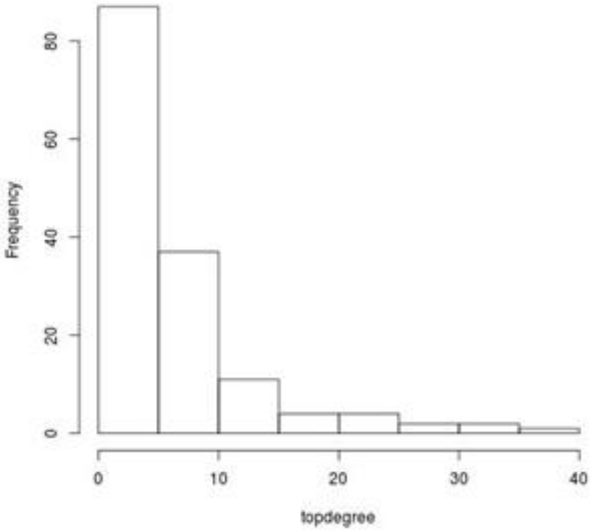

(a) Bigraph Gene Degree Distribution of BC10

ERR166336 (BC11)

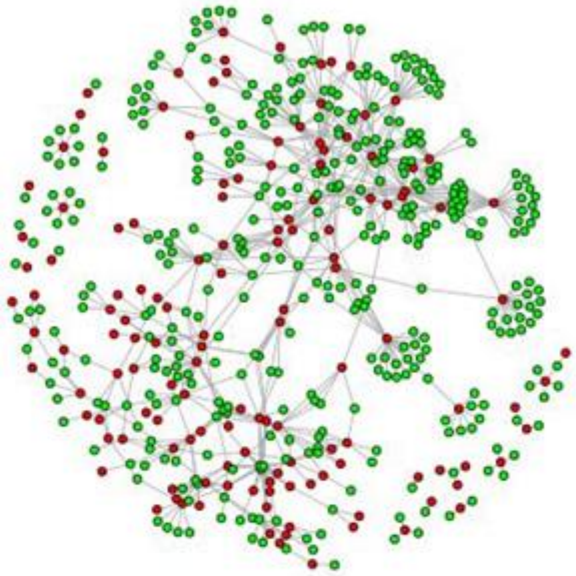

(a) Bipartite Pathway-Gene Interaction of BC11

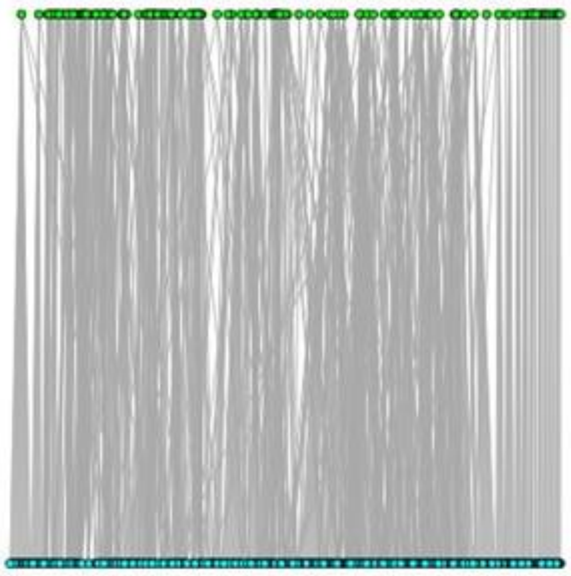

(a) Pathway-Gene Bipartite graph of BC11

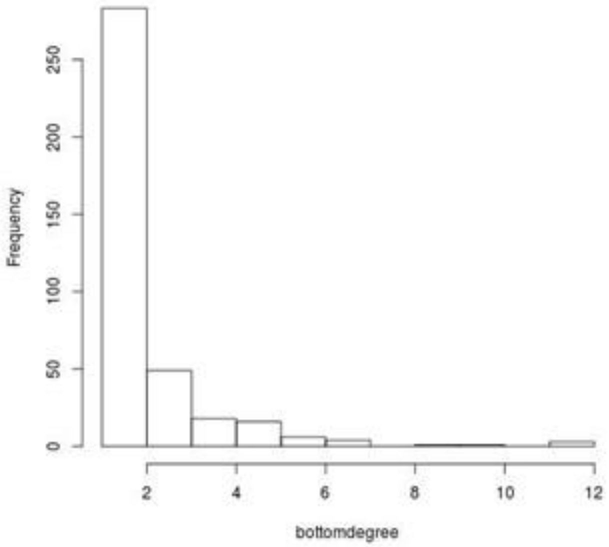

(a) Bigraph Pathway Degree Distribution of BC11

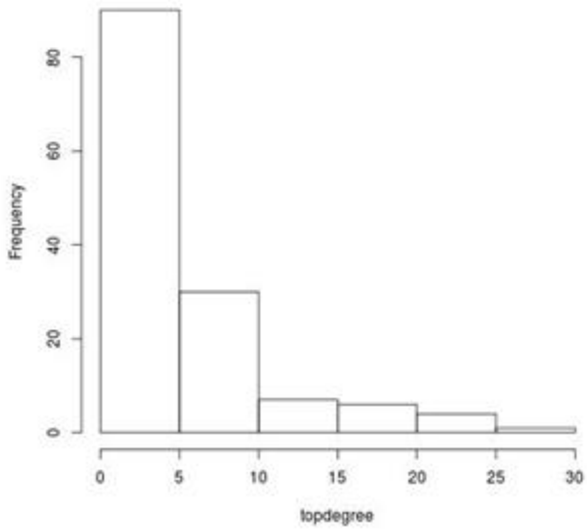

(a) Bigraph Gene Degree Distribution of BC11
