# Supplementary material for: XomAnnotate: Analysis of Heterogeneous and Complex Exome- A Step towards Translational Medicine
Source: PLoS One. 2015 Apr 23;10(4):e0123569. doi: 10.1371/journal.pone.0123569 (PMC4408095; doi:10.1371/journal.pone.0123569)

# S5_File

## Analysis of Pathway and Gene interaction networks

Results of graph theory based analysis of 11 non-BRCA1/BRCA2 breast cancer patients are presented here. It has included all the genes in KEGG cancer pathways that have been mutated in various samples. The central pathways and genes (betweenness) measured through graph theory are also represented for each breast cancer sample. It also shows the pathway-pathway and the gene-gene graphs that have been constructed for 11 cancer patients. It is observed that the purine metabolism (BC2, BC6, BC11) and propanoate metabolism (BC5, BC10) pathways are most commonly affected and RAF1 (BC2, BC6, BC11) and PRKCA (BC5, BC10) are affected central genes.

## <u>ERR166303 (BC1)</u>

Central Pathway = GLYCINE,_SERINE_AND_THREONINE_METABOLISM

Central Gene = SPHK2

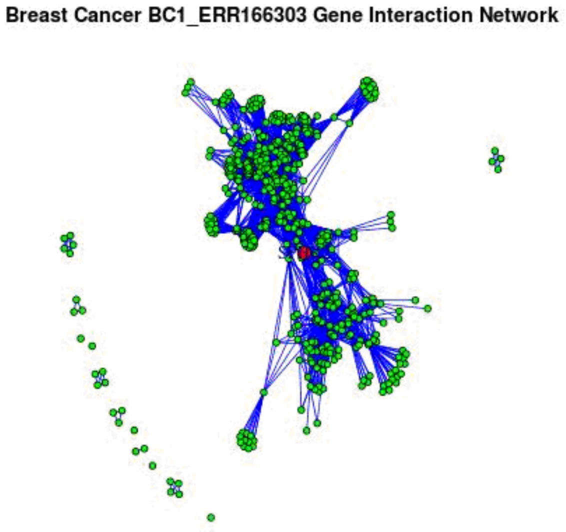


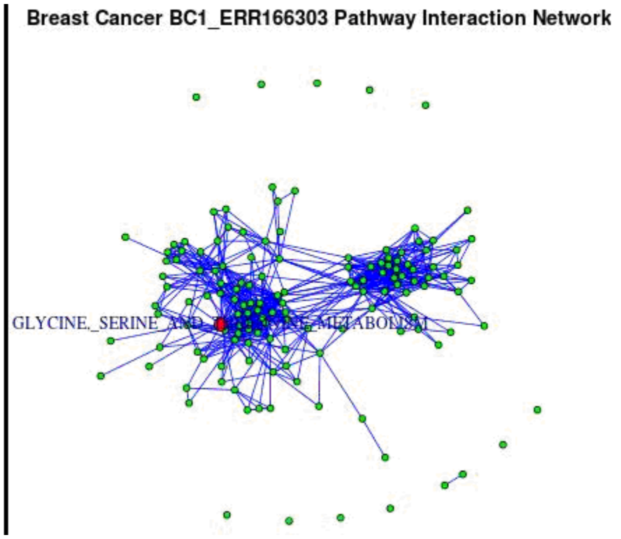

## ERR166304 (BC2)

Central Pathway = PURINE_METABOLISM

Central Gene = RAF1

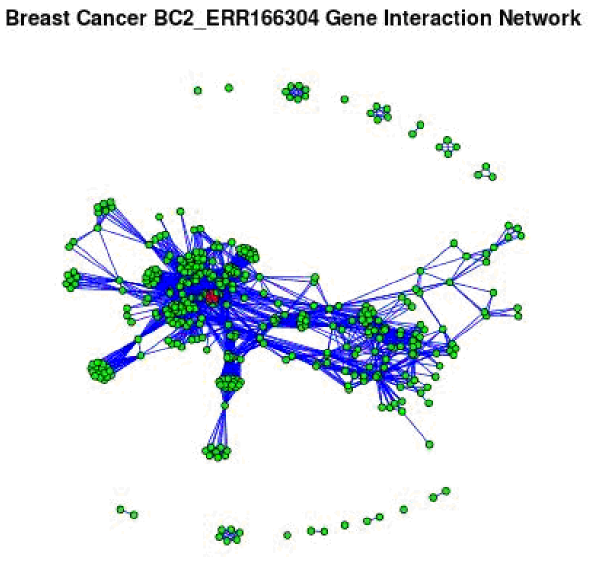


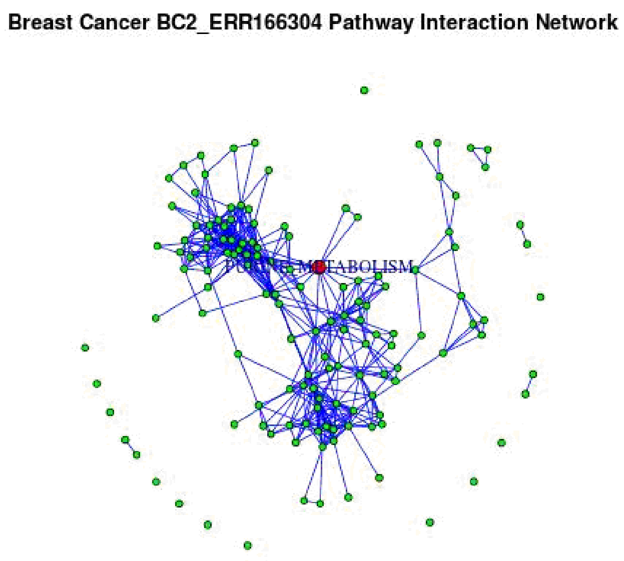


## ERR166307 (BC3)

Central Pathway = GLYCEROLIPID_METABOLISM

Central Gene = MET

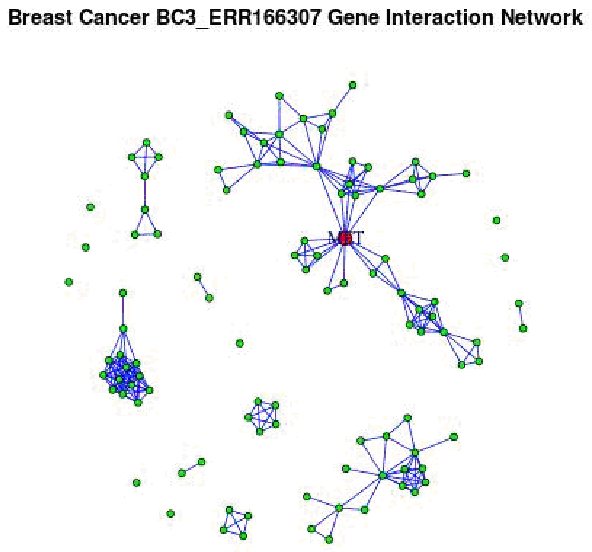


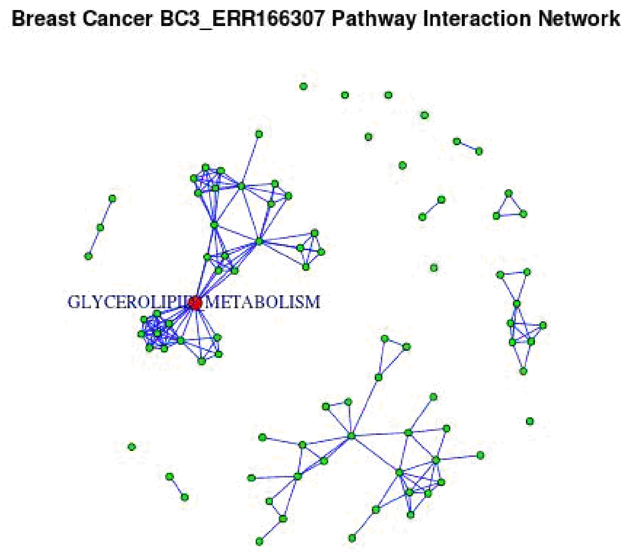

**ERR166308 (BC4)**

Central Pathway = FATTY_ACID_METABOLISM

Central Gene = PRLR

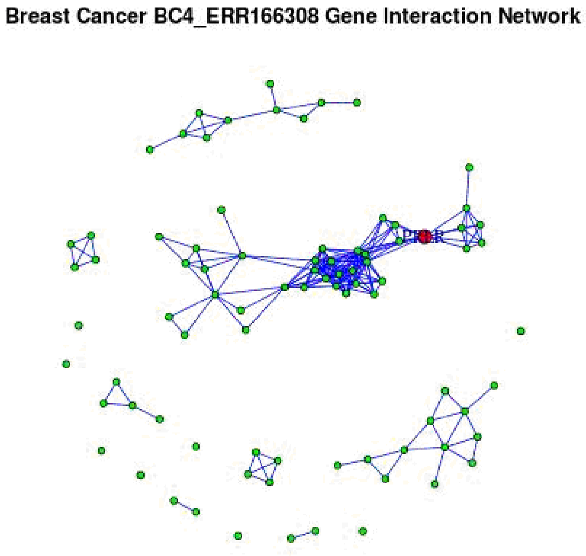


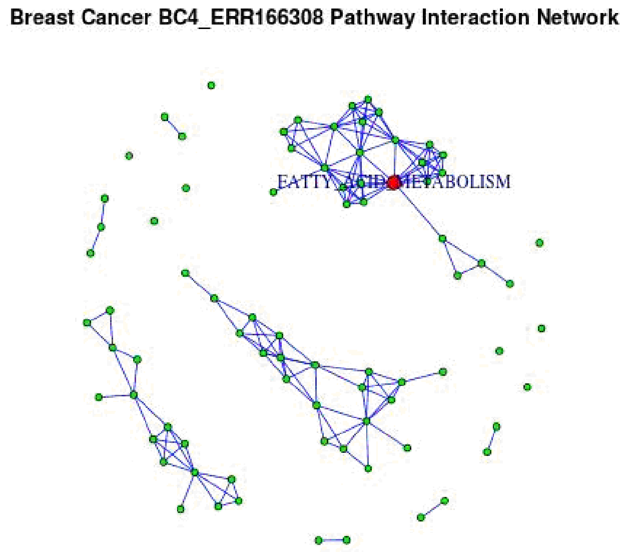


**ERR166310 (BC5)**

Central Pathway = PROPANOATE_METABOLISM

Central Gene = PRKCA

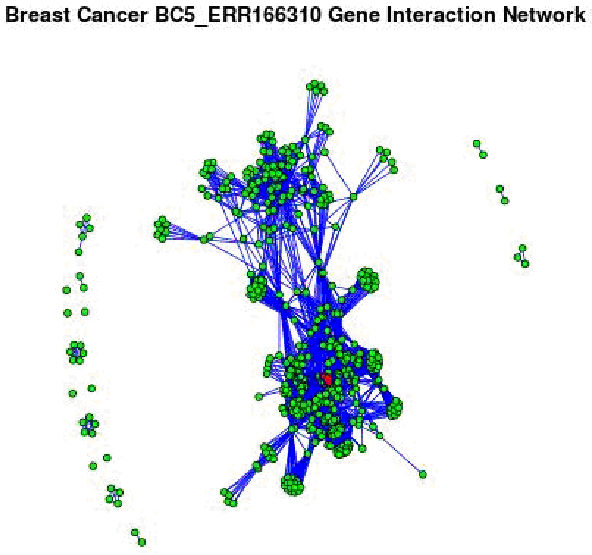


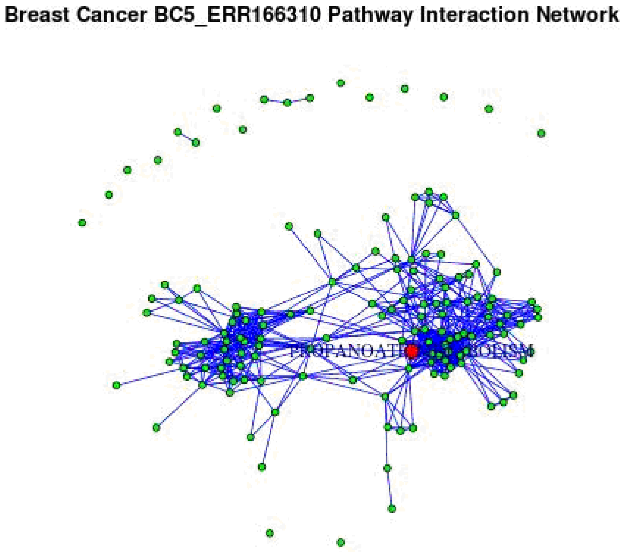

**ERR166312 (BC6)**

Central Pathway = PURINE_METABOLISM

Central Gene = RAF1

Breast Cancer BC6_ERR166312 Gene Interaction Network

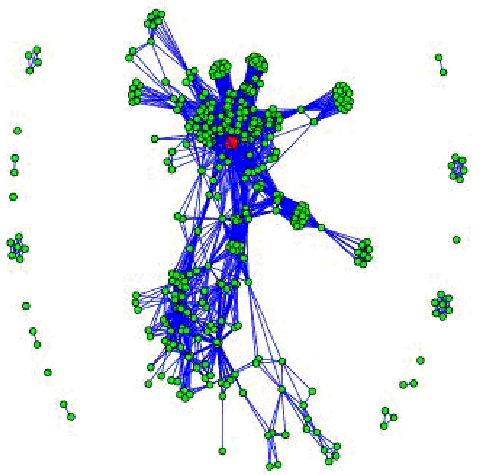

Breast Cancer BC6_ERR166312 Pathway Interaction Network

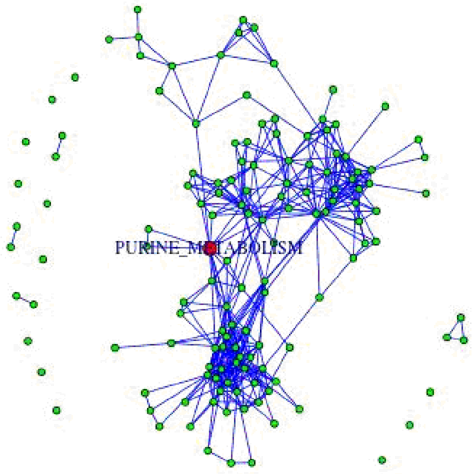


**ERR166315 (BC7)**

Central Pathway = APOPTOSIS

Central Gene = FASLG

Breast Cancer BC7_ERR166315 Gene Interaction Network

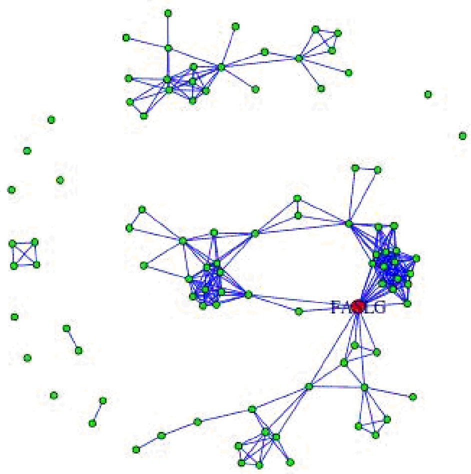


Breast Cancer BC7_ERR166315 Pathway Interaction Network

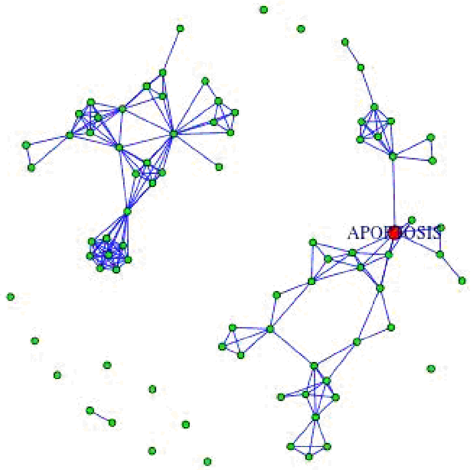

**ERR166330 (BC8)**

Central Pathway = INSULIN_SIGNALING_PATHWAY

Central Gene = SOS2

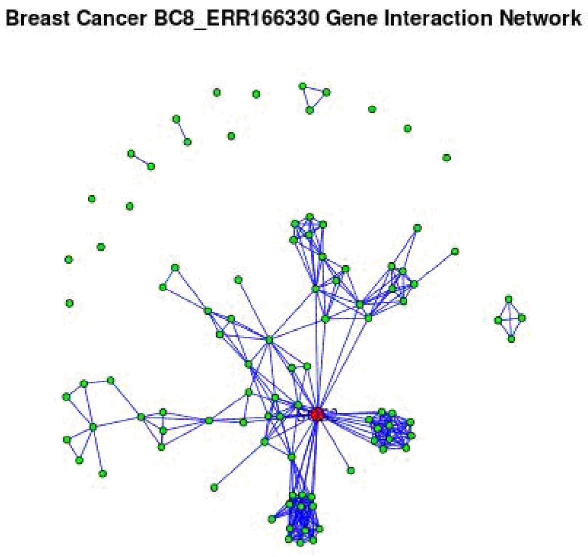


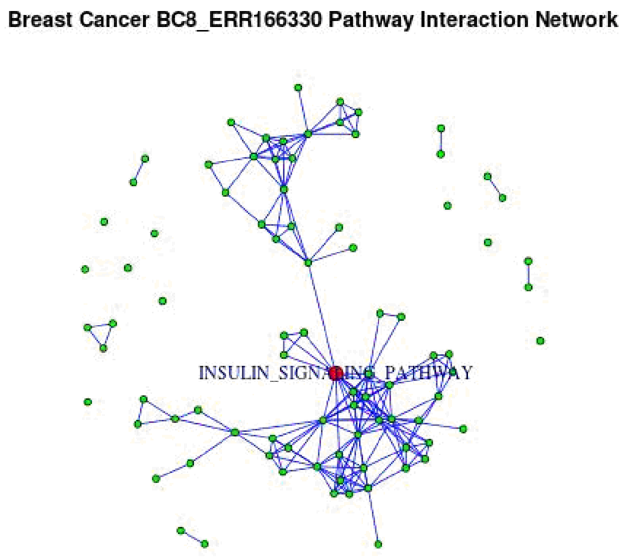


**ERR166333 (BC9)**

Central Pathway = ADHERENS_JUNCTION

Central Gene = EGFR

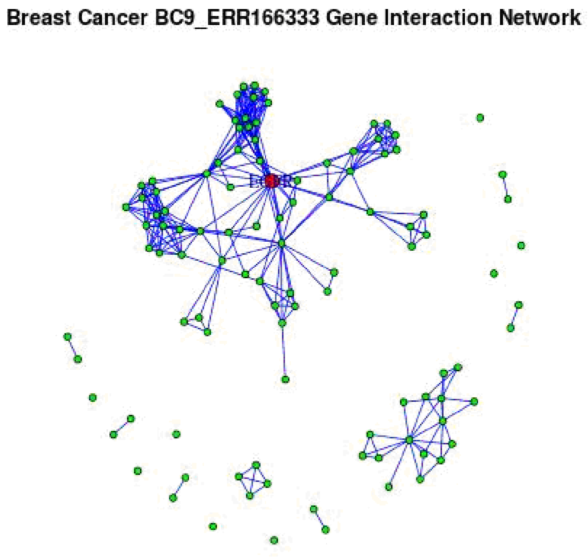


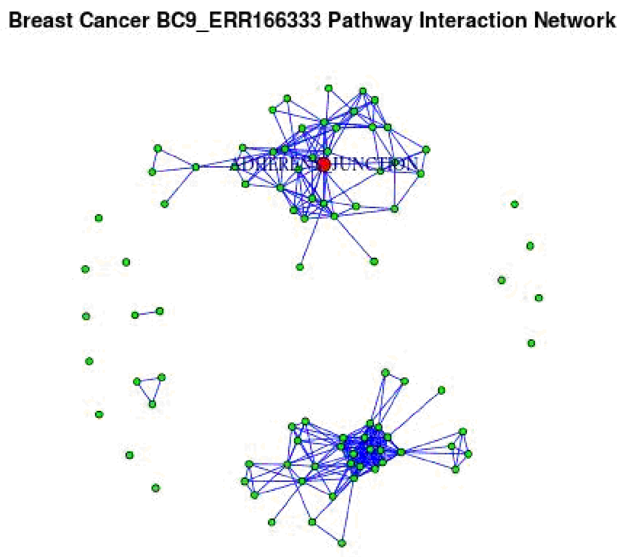

**ERR166335 (BC10)**

Central Pathway = PROPANOATE_METABOLISM

Central Gene = PRKCA

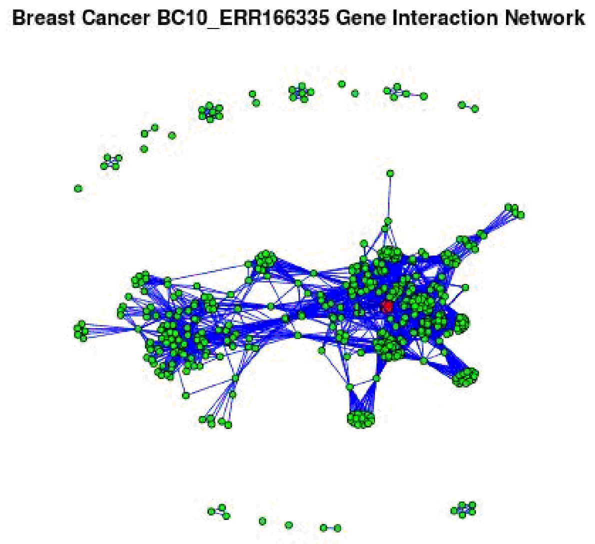


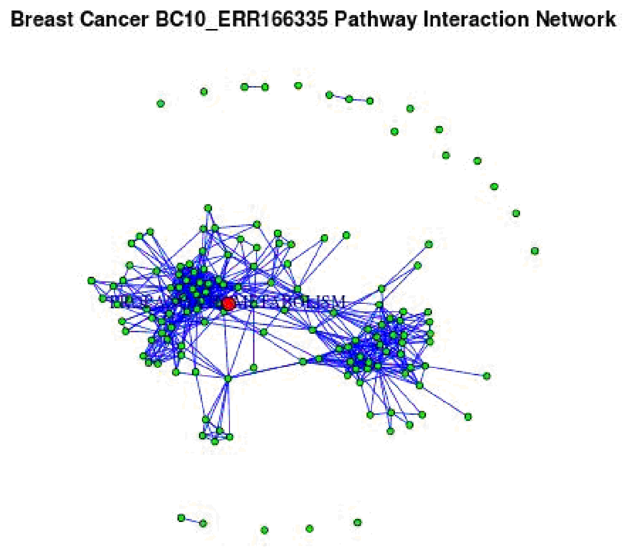


**<u>ERR166336 (BC11)</u>**

Central Pathway = PURINE_METABOLISM

Central Gene = RAF1

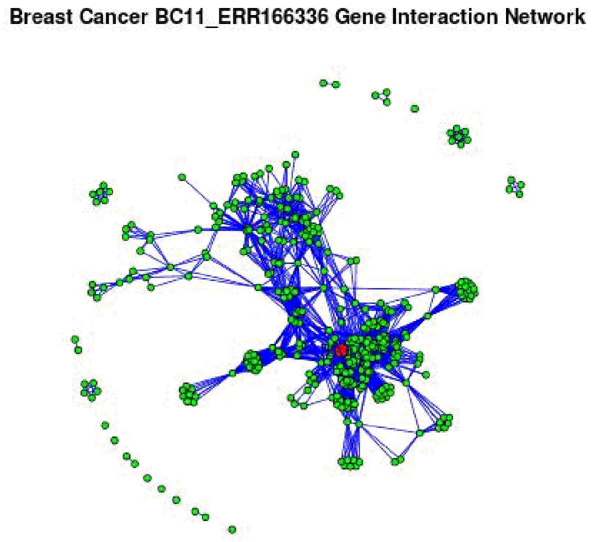


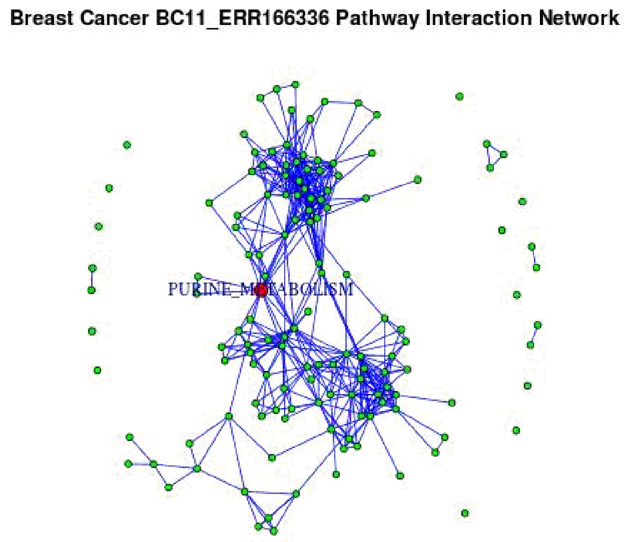

Supplement: S5 File — Results of graph theory based analysis of 11 non-BRCA1/BRCA2 breast cancer patients are presented here. It has included all the genes in KEGG cancer pathways that have been mutated in various samples. The central pathways and genes (betweenness) measured through graph theory are also represented for each breast cancer sample. It also shows the pathway-pathway and the gene-gene graphs that have been constructed for 11 cancer patients. It is observed that the purine metabolism (BC2, BC6, BC11) and propanoate metabolism (BC5, BC10) pathways are most commonly affected and RAF1 (BC2, BC6, BC11) and PRKCA (BC5, BC10) are affected central genes. (PDF) [file pone.0123569.s005.pdf]
