# Supplementary material for: XomAnnotate: Analysis of Heterogeneous and Complex Exome- A Step towards Translational Medicine
Source: PLoS One. 2015 Apr 23;10(4):e0123569. doi: 10.1371/journal.pone.0123569 (PMC4408095; doi:10.1371/journal.pone.0123569)
Supplement: S1 Table — This table gives information of the raw read count and number of variations detected (unfiltered) by the various tools like GATK’s Unified Genotyper, Freebayes, Delly, and Lumpy. (PDF) [file pone.0123569.s006.pdf]

S1_ Table

Downloaded exome data statistics: This table gives information of the raw read count and number of variations detected (unfiltered) by the various tools like GATK's Unified Genotyper, Freebayes, Delly, and Lumpy.

Sample Name	SRA ID	Number of reads in FASTQ format	Number of variants detected by GATK	Number of variants detected by Freebayes	Number of variants detected by Delly	Number of variants detected by Lumpy
**BC1**	ERR166303	31,781555	36,892	131,866	2,150	470,494
**BC2**	ERR166304	34,046,710	33,127	127,676	2,247	425,815
**BC3**	ERR166307	31,566,246	21,375	127,694	2,217	468,845
**BC4**	ERR166308	27,984,553	38,596	111,507	1,627	455,866
**BC5**	ERR166310	35,051,501	36,631	129,648	1,313	415,154
**BC6**	ERR166312	34,418,993	33,095	96,048	1,153	397,214
**BC7**	ERR166315	31,335,982	20,910	120,255	1,939	470,630
**BC8**	ERR166330	33,061,003	20,908	105,784	1,228	445,610
**BC9**	ERR166333	34,407,232	21,453	104,431	1,133	497,477
**BC10**	ERR166335	34,527,242	36,695	102,571	1,120	473,175
**BC11**	ERR166336	33,929,079	33,128	102,584	1,161	406,152
**H1**	ERR031613	30,392,863	20,804	204,243	203,600	582,408
**H2**	ERR031614	30,784,396	21,249	210,846	207,890	586,620
**H3**	ERR031615	34,188,883	21,843	475,983	4,678	476,041
**H4**	ERR031616	35,569,006	22,013	114,123	4,953	485,527
**H5**	ERR031617	31,903,427	20,996	108,304	6,249	507,784
**H6**	ERR031618	31,011,913	18,523	110,102	5,437	512,310
**H7**	ERR031619	25,566,841	19,464	223,033	175,928	505,912
**H8**	ERR031620	25,899,833	19,786	231,020	174,656	509,757
**H9**	ERR031621	23,342,501	16,795	173,149	3,687	676,347
**H10**	ERR031622	29,612,812	19,036	285,228	4,070	765,218
**H11**	ERR031624	29,711,398	20,635	165,244	176,397	572,143
**H12**	ERR031625	28,969,101	20,945	200,421	171,711	575,888
**H13**	ERR031626	26,885,917	20,628	185,367	162,736	555,543
