# Supplementary material for: XomAnnotate: Analysis of Heterogeneous and Complex Exome- A Step towards Translational Medicine
Source: PLoS One. 2015 Apr 23;10(4):e0123569. doi: 10.1371/journal.pone.0123569 (PMC4408095; doi:10.1371/journal.pone.0123569)
Supplement: S2 Table — The table gives detailed information about the samples used, distribution of variations detected by the various tools, the total number of variations considered for meta-analysis and the total number of KEGG pathway genes affected. (PDF) [file pone.0123569.s007.pdf]

S2_Table

Detailed inventory of samples used for analysis: The table gives detailed information about the samples used, distribution of variations detected by the various tools, the total number of variations considered for meta-analysis and the total number of KEGG pathway genes affected.

Total Variations (SNP + SV)	Effected genes in KEGG Cancer Pathways
3,656	487
2,858	380
727	101
650	80
3,790	464
2,844	377
740	94
782	96
828	102
3,638	459
2,844	376
3,325	414
3,621	429
928	129
868	118
1,111	158
609	74
2,027	252
2,046	264
466	54
663	77
3,051	374
2,763	344
2,437	276
